# Disruption of *grin2B*, an ASD-associated gene, produces social deficits in zebrafish

**DOI:** 10.1186/s13229-022-00516-3

**Published:** 2022-09-22

**Authors:** Josiah D. Zoodsma, Emma J. Keegan, Gabrielle R. Moody, Ashwin A. Bhandiwad, Amalia J. Napoli, Harold A. Burgess, Lonnie P. Wollmuth, Howard I. Sirotkin

**Affiliations:** 1 Graduate Program in Neuroscience, Stony Brook University, Stony Brook, NY 11794-5230 USA; 2 Department of Neurobiology and Behavior, Stony Brook University, Stony Brook, NY 11794-5230 USA; 3 Graduate Program in Molecular and Cellular Pharmacology, Stony Brook University, Stony Brook, NY 11794-5230 USA; 4 Department of Biochemistry and Cell Biology, Stony Brook University, Stony Brook, NY 11794-5230 USA; 5 Center for Nervous System Disorders, Stony Brook University, Stony Brook, NY 11794-5230 USA; 6 Division of Developmental Biology, Eunice Kennedy Shriver National Institute of Child Health and Human Development, Bethesda, MD USA

**Keywords:** NMDA receptors, GluN2B, Autism spectrum disorders, Social behaviors

## Abstract

**Background:**

Autism spectrum disorder (ASD), like many neurodevelopmental disorders, has complex and varied etiologies. Advances in genome sequencing have identified multiple candidate genes associated with ASD, including dozens of missense and nonsense mutations in the NMDAR subunit GluN2B, encoded by *GRIN2B*. NMDARs are glutamate-gated ion channels with key synaptic functions in excitatory neurotransmission. How alterations in these proteins impact neurodevelopment is poorly understood, in part because knockouts of GluN2B in rodents are lethal.

**Methods:**

Here, we use CRISPR-Cas9 to generate zebrafish lacking GluN2B (*grin2B*^*−/−*^). Using these fish, we run an array of behavioral tests and perform whole-brain larval imaging to assay developmental roles and functions of GluN2B.

**Results:**

We demonstrate that zebrafish GluN2B displays similar structural and functional properties to human GluN2B. Zebrafish lacking GluN2B (*grin2B*^*−/−*^) surprisingly survive into adulthood. Given the prevalence of social deficits in ASD, we assayed social preference in the *grin2B*^*−/−*^ fish. Wild-type fish develop a strong social preference by 3 weeks post fertilization. In contrast, *grin2B*^*−/−*^ fish at this age exhibit significantly reduced social preference. Notably, the lack of GluN2B does not result in a broad disruption of neurodevelopment, as *grin2B*^*−/−*^ larvae do not show alterations in spontaneous or photic-evoked movements, are capable of prey capture, and exhibit learning. Whole-brain imaging of *grin2B*^*−/−*^ larvae revealed reduction of an inhibitory neuron marker in the subpallium, a region linked to ASD in humans, but showed that overall brain size and *E*/*I* balance in *grin2B*^*−/−*^ is comparable to wild type.

**Limitations:**

Zebrafish lacking GluN2B, while useful in studying developmental roles of GluN2B, are unlikely to model nuanced functional alterations of human missense mutations that are not complete loss of function. Additionally, detailed mammalian homologies for larval zebrafish brain subdivisions at the age of whole-brain imaging are not fully resolved.

**Conclusions:**

We demonstrate that zebrafish completely lacking the GluN2B subunit of the NMDAR, unlike rodent models, are viable into adulthood. Notably, they exhibit a highly specific deficit in social behavior. As such, this zebrafish model affords a unique opportunity to study the roles of GluN2B in ASD etiologies and establish a disease-relevant in vivo model for future studies.

**Supplementary Information:**

The online version contains supplementary material available at 10.1186/s13229-022-00516-3.

## Background

Autism spectrum disorder (ASD) is a neurodevelopmental disorder affecting upwards of 1 in 100 children [1–3]. ASD is characterized by impairments in social skills, sensory processing, and communication as well as stereotypic behaviors [4]. Symptoms typically present in early childhood and effective therapies are lacking [5, 6]. Causal mechanisms underlying changes in early brain development leading to ASD are not well defined. The genetic causes of ASD are exceptionally diverse, and hundreds of candidate loci have been identified [7–11]. Many ASD-associated genes are expressed in both developing excitatory and inhibitory neurons and play known roles in synaptic transmission [11]. Given the complex etiology of ASD, a deeper understanding of the role of these ASD-associated genes in early development is needed.

NMDA receptors (NMDARs) are glutamate-gated, calcium-permeable ion channels that mediate a slow component of glutamatergic transmission [12]. They play key roles in early brain development [13] and the plasticity underlying higher-order processes [14, 15]. NMDARs are obligate heterotetramers, comprised of two GluN1 subunits and typically some combination of GluN2(A-D) subunits [12]. The various GluN2 subunits impart distinct functional and cell biological properties to the receptor and play myriad roles in brain development. Accordingly, mutations in NMDAR subunits are associated with numerous neurodevelopmental disorders including ASD, epilepsy, intellectual disability, and schizophrenia [16–18].

*GRIN2B*, which encodes the GluN2B subunit, is a high-confidence ASD gene [8, 11, 19]. GluN2B is highly expressed in humans during early brain development and has critical functions in neurogenesis and circuit formation [20–22]. The complex activities of GluN2B in early development remain incompletely defined, in part because murine knockouts of GluN2B do not survive past perinatal stages [23]. As a highlight of this complexity, mutations causing both loss and gain of GluN2B function are associated with ASD as well as other neurodevelopmental disorders [24, 25].

Here, we investigate the validity and robustness of the zebrafish model to study GluN2B in development. This system affords detailed analysis of the role of GluN2B in early brain development and the emergence of complex behaviors, offering numerous readouts of the developing nervous system. Additionally, zebrafish are highly social creatures [26], facilitating the study of the role of GluN2B in social preference development. In zebrafish, the GluN2B subunit is encoded by two paralogous genes: *grin2Ba* and *grin2Bb* [27]. We find that fish with frameshift mutations in both *grin2B* paralogues, and thus lacking all GluN2B, survive into adulthood and are fertile. This offers a unique opportunity to study the developmental roles of GluN2B and characterize its requirements in brain development and the formation of various zebrafish behaviors. We find that zebrafish lacking GluN2B show social deficits, despite having no significant changes in whole-brain size, glutamatergic neuron density, or overall excitatory/inhibitory (*E*/*I*) balance. Nevertheless, we find significant reduction in a marker of inhibitory neurons in a small subset of regions, most notably the subpallium, an area containing subcortical structures whose dysfunction is associated with ASD phenotypes [28, 29].

## Materials and methods

### Zebrafish maintenance

Adult zebrafish strains were fed a diet of artemia and GEMMA micropellets and maintained at 28.5 °C under a 13:11 h light-to-dark cycle. The wild-type strain used for all experiments was a hybrid wild-type background consisting of Tüpfel long fin crossed to Brian’s wild type. The experiments and procedures were approved by the Stony Brook University Institutional Animal Care and Use Committee.

### Whole-cell electrophysiology

Human embryonic kidney 293 (HEK293) cells were maintained and grown as previously described [30, 31]. Zebrafish cDNA constructs were commercially synthesized (GenScript) in a pcDNA3.1(1)-p2A-eGFP vector. Human GluN1 (hGluN1) and either human GluN2B (hGluN2B) or zebrafish GluN2Bb (zGluN2Bb) cDNA constructs were co-transfected into HEK293 cells along with a separate peGFP-Cl construct at a ratio of 4:4:1 (N1:N2:eGFP) for whole-cell recordings. After transfection, cells were bathed in medium containing the GluN2 competitive antagonist DL-2-amino-5-phosphopentanoic acid (100 mM, Tocris Bioscience) and Mg2+ (100 mM). All experiments were performed 24–48 h after transfection. Whole-cell currents were recorded at room temperature (20–23 °C) as previously described [30–32]. Patch microelectrodes were filled with an intracellular solution (in mM as follows): 140 KCl, 10 HEPES, 1 BAPTA, 4 Mg21-ATP, 0.3 Na1-GTP, pH 7.3 (KOH), 297 mOsm (sucrose). Our standard extracellular solution consisted of the following (mM): 150 NaCl, 2.5 KCl, and 10 HEPES, pH 7.2 (NaOH). Currents were measured within 10 min of going whole cell. External solutions were applied using a piezo-driven double-barrel application system. For display, NMDAR currents were digitally refiltered at 500 Hz and resampled at 1 kHz.

### CRISPR-Cas9 gene targeting and genotyping

gRNAs were designed through the IDT custom gRNA Design Tool. gRNAs were complexed to Cas9 protein, to form a ribonucleoprotein, using the Alt-R CRISPR-Cas9 System (IDT); 0.5 nl of ribonucleoprotein (25 pg of gRNA and 125 pg of Cas9 protein) was injected into the cell of pronased, 1-cell wild-type embryos. 20–25 injected embryos for each gRNA were assayed by PCR for lesions using primers flanking the gRNA target site. Those siblings, in which lesions were detected in the F0, were grown into adulthood to be assayed for germline transmission. To minimize the effects of potential off-target endonuclease activity, all lines were outcrossed at least twice prior to assaying behavior in any paradigm. Individual mutations were isolated and sequenced using Sanger Sequencing. Ensembl gene IDs for *grin2Ba* (ENSDARG00000079348) and *grin2Bb* (ENSDARG00000030376).

Primers used for both initial screen and subsequent genotyping:

- *grin1a*—F (TGGGCTGGCTTCGCTATGAT) and R (GGGTCGTTGATGCCGGTGAT)
- *grin1b—*F (GGTGCCCCTCGGAGCTTTTC) and R (GGAAGGCTGCCAAATTGGCAGT)
- *grin2Aa—*F (AGACCCCCACGGACCGTCTTTC) and R (CGTGCCTTTGGGGTTTTGCACA)
- *grin2Ab—*F (GCTCACTCCTCCGCACTAACTT) and R (GACCCCACAGCAGCCAGACA)
- *grin2Ba—*F (TGGTCTCCGTCTGGGCCTTC) and R (GACACCTGGTCCACGTACTCCT)
- *grin2Bb—*F (CTATTTGGCTTCTTTGGGGTTTGGT) and R (CGAAGAAGGCCCAGACCGAAA)
- *grin2Da—*F (AAGACGTGTCTGTGTTTTCTTCTG) and R (CGGAGTTATTAAACACCAGAGC)
- *grin2Db—*F (TTCTGTGGGCTCTGGTCTTT) and R (TAGCTGGCCAGGAAGATGAC)
- *fmr1—*F (GAATATGCAGCCTGTGATGCCACCCTAAATGAAATCGTCACATTAGAGAGGGTA) and R (TTGGCCAAACTCCATGACATCCTG) followed by a digest with RSAI

### rt-PCR

RNA was extracted from pools of either 4 larvae at 3 dpf or 2 larvae at 5 dpf, in 200 uL of TRIzol (Invitrogen) and isolated with Direct-zol RNA Miniprep (Zymo Research). cDNA was synthesized from 200 ng of RNA using Superscript II Reverse Transcriptase (Invitrogen) and analyzed using the following primers:

- *grin2Ba—*F (TGGTCTCCGTCTGGGCCTTC) and R (GAAGCGAAACGGAGGAGAG)
- *grin2Bb—*F (CTGTCGGCTATAATCGCTGC) and R (CGAAGAAGGCCCAGACCGAAA)

### Zebrafish behavior assays

Zebrafish were grown in 150 mm Petri dishes at densities of less than 100 per plate. To minimize any effects of genetic background on the behavior, larvae were assayed from multiple clutches from different parents. Genotypes were acquired post hoc for all assays, as outlined above, to ensure experimenters were blind to genotype.

#### Social behavior assay

Larvae were grown in Petri dishes until 5 dpf and were transferred to juvenile cylinders with densities between 35 and 50 per cylinder until the assays were performed at 1, 2, 3, or 4 wpf. Juvenile zebrafish were placed in a lane of a social behavior chamber [33] containing system water prior to the assay. The social behavior chamber was placed into a Zantiks MWP unit (Zantiks) held at a temperature of 28 °C, and fish were allowed to acclimate for 5 min. Movement of test fish was recorded for 20 min, and social preference index was calculated through the following formula:

$$\mathrm{SPI}= \frac{\left(1*{T}_{Z4}\right)+\left(0.5*{T}_{Z3}\right)+\left(-0.5*{T}_{Z2}\right)+(-1*{T}_{Z1})}{{T}_{\mathrm{total}}}$$

where $\left({T}_{Z\#}\right)$ represents the time spent in zones 1–4. All assays were run with control fish from relative clutches (either homozygous intercrosses or heterozygous intercrosses).

#### Spontaneous and photic-evoked behavior

Spontaneous and photic-evoked assays were performed as previously reported [34].

#### Prey capture feeding assay

Prey capture assays using paramecia were performed as previously reported [34]. Larval prey capture learning assays were adapted from [33]. At 5 and 6 dpf, fish in the naïve group were fed fish flakes (Hikari USA Inc.), while fish in the experienced group were fed Paramecium Caudatum (Carolina Biological Supply). Feedings occurred for 6 h each day; food was refreshed and media washed after 3 h. Prey capture paramecia feeding experiments were run at 7 dpf, as previously reported [34], aside for substituting Paramecium Multimicronucleatum for Paramecium Caudatum.

### qPCR

cDNA was generated from RNA extracted from pools of either 4 larvae at 3 dpf or 2 larvae at 5 dpf as outlined above. qPCR assay was run using PerfeCTa SYBR Green FastMix (QuantaBio) on a LightCycler480 (Roche Diagnostics). Transcript levels were obtained from averaged biological replicates for each sample and were normalized to ß-actin. Primers utilized in these experiments:

- *grin1a*—F (ATAAAGACGCCCGCAGGAAGC) and R(CGTGCTGACAGACGGGTCCGAC);
- *grin1b—*F (AATGCAGCTGGCCTTTGCAGC) and R(CTCTTGATGTTGGAGGCCAGGTTG)
- *grin2Aa—*F (GCTTCTGCATTGACATCCTCA) and R(AACGCTCCTCATTGATTGTCAG)
- *grin2Ab—*F (AGACATCCTGAAGAAGATCGC) and R(CTCTGACCGCTCTTCATTTATG)
- *grin2Ba—*F (CATCAGTGTGATGGTGTCCAG) and R(CACAGGACTGAAGTACTCGAAG)
- *grin2Bb—*F (GTCTGTCAAATTCACCTACGATC) and R(GCACTGAGAAGTCAATGACCT)
- *grin2Ca—*F (GAGCGTTCAGAGATCATTGACT) and R(CACGACCGTAAGACACATGAC)
- *grin2Cb—*F (TCCTAAAGAAGCTCTCACGCA) and R(TGAGCGCTCCTCGTTGATG)
- *grin2Da—*F (CAGAGGTTGTGGAGTTCTCTG) and R(GCACTGAGAAGTCAATGACCT)
- *grin2Db—*F (GTTTCTGCATCGACATCCTCA) and R(CGTCAGGCACATCACAAAC)

### Whole-brain confocal scans

*grin2B*^*−/−*^ fish were crossed to fish heterozygous for both *grin2B* mutations and containing two transgenic lines: Tg(*dlx6a-1.4kbdlx5a/dlx6a:GFP)* [35] and Tg(*vglut:DsRed*) [36]*.* Genotypes were acquired post hoc distinguishing *grin2B* controls from *grin2B*^*−/−*^ fish. 6 dpf larvae were placed in PTU (30 mg/L) at 1 dpf. Larvae positive for both transgenes were mounted in 1.5% low melting point agarose in a Lab-Tek II Chambered Coverglass. Brain images were acquired on an Olympus FV-1000 confocal microscope at a magnification of 30×, through a *Z*-range of 350 microns, at 2-micron increments. Laser intensity was adjusted for each sample throughout the scan to adjust for loss in deeper layers, with the same intensity changes occurring in each sample at equivalent depths relative to the dorsal-most plane of the brain. Two image sets were acquired for each brain, one encompassing the anterior to middle regions and the other encompassing the middle to posterior extent. These image sets were stitched in ImageJ using the Pairwise Stitching plug-in [37]. Stitched images were registered utilizing the Zebrafish Brain Browser, and analysis of registered brains was done through the Cobra Z pipeline [38]. Full-brain visualization and coronal sections were generated using the Zebrafish Brain Browser [39]. Measurement of mean intensity in subdivisions of the subpallium used ROIs previously computationally defined from expression data [40].

### Experimental design and statistics

Data analyses were performed using IgorPro, Excel, RStudio, and MiniTab 18. Average values are presented as mean ± SEM except for boxplots which indicate the median, quartiles, and range. For experiments where a control was compared to an individual mutant group, unpaired two-tailed Student’s *t* test was used to test for significance. For comparisons of multiple groups, a one-factor ANOVA, followed with a post hoc Tukey’s test was used. A two-factor ANOVA was used when looking at both genotype and time as variables. Estimation plots were generated using Python and DABEST [41]. Statistical significance was set at *p* < 0.05.

Sex in zebrafish is indeterminate at the age of the larval and juvenile behavior assays in this study and was thus not a factor in experimental design.

## Results

### Zebrafish GluN2B orthologues share high sequence and domain homology with human GluN2B

Each NMDAR subunit has two zebrafish paralogues that show high degrees of sequence similarity with their human counterparts [27]. To assess the relevance of studying zebrafish GluN2B as a model of human disease, we analyzed the structural and functional similarities between the human GluN2B (hGluN2B, encoded by *GRIN2B*) and the zebrafish paralogues (zGluN2Ba and zGluN2Bb, encoded by *grin2Ba* and *grin2Bb,* respectively).

**Fig. 1:**
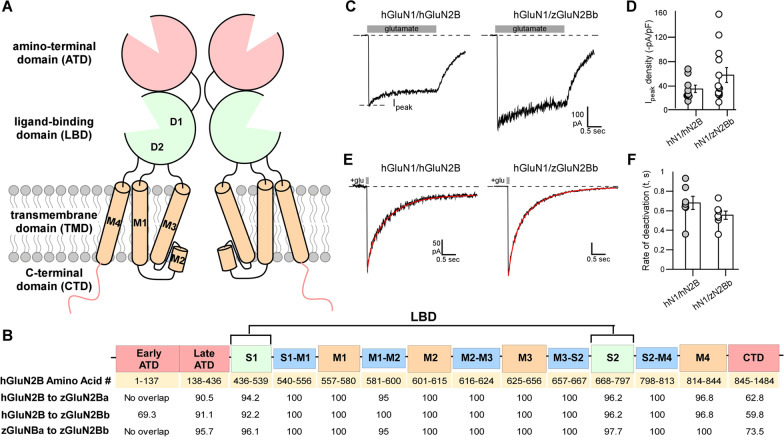
Zebrafish orthologues have a similar sequence and function to human GluN2B (hGluN2B) **A** Schematic depiction of membrane topology of two NMDAR subunits. Functional NMDARs are tetramers composed of two obligatory GluN1 subunits and typically two GluN2 (**A**–**D**) subunits. NMDARs are composed of four modular domains: the extracellular ATD and LBD; the membrane-embedded TMD; and the intracellular CTD. These domains are intrinsic to the full-length receptor as well as individual subunits. Each individual subunit contributes three transmembrane segments (M1, M3, and M4) and a M2 pore loop to form the ion channel. **B** Linear representation of an NMDAR subunit. Colors match the 3D cartoon in (**A**) with TMD-associated linkers indicated in blue. The S1 and S2 combine to generate the LBD, which in the 3-dimensional structure is referred to as D1 (mainly composed of S1) and D2 (mainly composed of S2). Table, broken down by NMDAR region, showing the amino acid alignment between hGluN2B and each of the zebrafish GluN2B paralogues (zGluN2Ba and zGluN2Bb). Values indicate percent similarity of amino acids. **C** Whole-cell currents from HEK 293 cells expressing human GluN1 subunit (hGluN1), with either hGluN2B (hGluN1/hGluN2B) (left panel) or the zGluN2Bb (hGluN1/zGluN2Bb) (right panel) Currents were elicited by a 2.5 s application of glutamate (1 mM, gray bar) in the continuous presence of glycine (0.1 mM). Holding potential, − 70 mV. **D** Variations in peak current amplitudes between the constructs. Bar graph (mean ± SEM) showing peak current amplitude density (*I*_peak_/Membrane capacitance). Circles are individual recordings. Number of recordings are 11 and 12 (left to right). **E** Whole-cell currents in response to brief (2 ms) glutamate applications, as occurs at synapses. **F** Bar graph (mean ± SEM) showing deactivation rates. Values were not significantly different. Number of recordings are 11 and 12 (left to right)

All glutamate receptors share a common topology consisting of an amino-terminal (ATD) and a ligand-binding (LBD) domain, both located extracellularly, a transmembrane domain (TMD) embedded in the membrane forming the ion channel, and an intracellular C-terminal domain (CTD) (Fig. 1A). The required components for ion channel function are the LBD and the TMD. Zebrafish GluN2B paralogues share high protein sequence similarity to each other, with > 95% similarity in the LBD and TMD (Fig. 1B). Notably, the human orthologue, hGluN2B, shares greater than 90% amino acid similarity with each zebrafish paralogue in all domains except the early ATD and CTD (Fig. 1B). Each component of the LBD and TMD also contain the same number of amino acids, highlighting the conservation of protein domains both between species and between paralogues (Additional file 1: Fig. S1). Hence, zebrafish paralogues presumably have the same basic structure as their mammalian counterparts.

### Zebrafish GluN2B can form ion-conducting channels with human GluN1

Functional NMDARs require co-expression of GluN1 and GluN2 subunits. To compare the physiological properties of zebrafish and human GluN2B, we co-transfected either human GluN2B (hGluN2B) or zebrafish GluN2Bb (zGluN2Bb) along with human GluN1 (hGluN1) into a heterologous expression system, HEK293 cells. Zebrafish GluN1 paralogues (zGluN1a and zGluN1b) form Ca^+2^-permeable channels with mammalian GluN2 subunits [34]. Similarly, we found that glutamate applications to HEK293 cells transfected with hGluN1 and zGluN2Bb, evoked inward currents (Fig. 1C) whose magnitude, on average, was indistinguishable from that observed in hGluN1/hGluN2B receptors (Fig. 1D).

A key functional feature of GluN2B-containing NMDARs, as compared to GluN2A-containing receptors, is a slow deactivation rate in response to brief synaptic-like glutamate applications [15, 42]. To test whether NMDAR containing zGluN2Bb showed slow deactivation kinetics, we applied brief (2 ms) glutamate applications to hGluN1/hGluN2B or hGluN1/zGluN2Bb receptors (Fig. 1E). We detected no significant difference in deactivation rates between hGluN2B and zGluN2Bb-containing NMDARs (Fig. 1F). Hence, zGluN2Bb replicates a key functional feature of human GluN2B-containng NMDARs. Together, the sequence and functional similarities between zebrafish and human GluN2B suggest zebrafish are an appropriate model to study the developmental functions of GluN2B.

### Generation of zebrafish *grin2Ba *and *grin2Bb* loss-of-function mutations

**Fig. 2:**
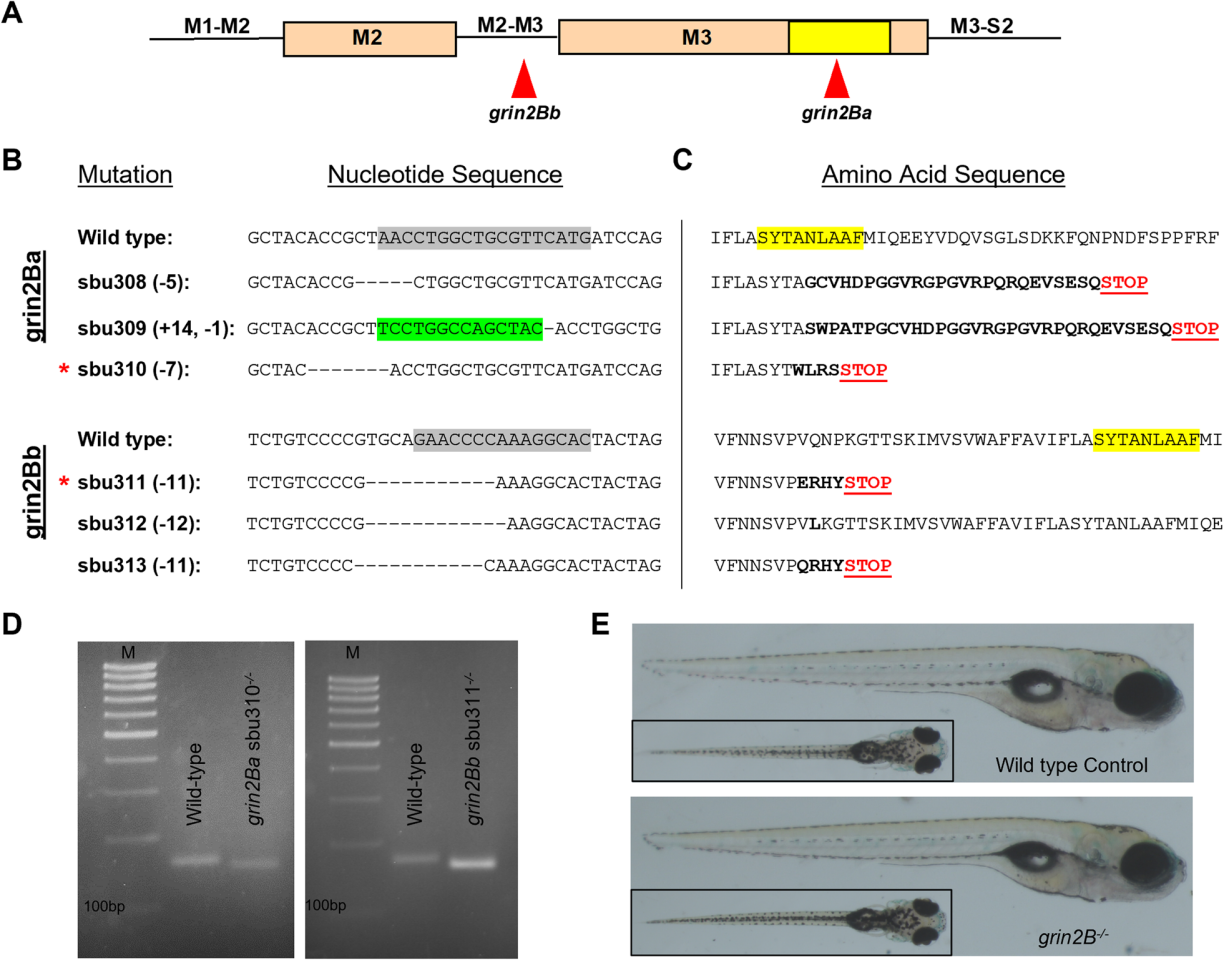
Generation of loss-of-function lesions in *grin2Ba* and *grin2Bb* using CRISPR-Cas9. **A** Linear representation of the M2 and M3 segments indicating gRNA target sites for *grin2Ba* and *grin2Bb*. The SYTANLAAF motif (indicated in yellow) is the most highly conserved motif in iGluRs and is fundamental to their function. **B** and **C** Alignments of **B** nucleotide and **C** amino acid sequences. gRNA target sites on the nucleotide sequence are indicated (gray highlights). Induced mutations within the nucleotide sequences are denoted as either dashes (deletions) or highlighted green (insertions). Altered amino acid sequences (bolded) and early stop codons (Red STOP) are generated in all alleles except sbu312. Such early translation termination events would prevent encoding the SYTANLAAF motif (yellow highlight) as well as the D2 lobe of the LBD, which would make the receptor non-functional. **D** Only mutant mRNA is detected for each *grin2B* lesion by rt-PCR. cDNA amplification of: *grin2Ba*^+/+^ and *grin2Ba*^−/−^ (unless otherwise noted, *grin2Ba*^−/−^ denotes the sbu310 allele) producing expected product sizes of 158 bp and 151 bp, respectively; and *grin2Ba*^+/+^ and *grin2Bb*^−/−^ (unless otherwise noted, *grin2Bb*^−/−^ denotes the sbu311 allele) producing expected product sizes of 171 bp and 160 bp, respectively. M denotes marker. For all genotypes, RNA was collected from homozygous intercrosses at 5 dpf. **E** Lateral and dorsal (inlet) images of representative 6 dpf wild type (top) and *grin2B*^*−/−*^ (bottom)

To study GluN2B in zebrafish development, we disrupted the *grin2Ba* and *grin2Bb* genes using CRISPR-Cas9 [43, 44]. gRNA target sites were designed to disrupt the highly conserved SYTANLAAF motif, found in the M3 transmembrane helix (Fig. 2A) [12, 45]. We generated 3 germline mutations in each gene (Fig. 2B). Frameshifts and early stop codons were generated in all but one allele (Fig. 2C), and the alleles generating the earliest stop codons, sbu310 (*grin2Ba*) and sbu311 (*grin2Bb*), were selected for further study. Both alleles yielded only mRNA transcripts harboring the deletions (Fig. 2D). Given that the stop codon occurred prior to the end of the SYTANLAAF motif, these mutant transcripts would yield proteins lacking an intact pore, a functioning ligand-binding domain (due to the lack of S2), and no M4 transmembrane segment, which would make any generated protein non-functional (Fig. 1B).

### Zebrafish lacking GluN2B are viable

Zebrafish homozygous mutants for either *grin2Ba* or *grin2Bb* are viable and survive in Mendelian ratios into adulthood (data not shown). To test the developmental requirement for GluN2B, we generated *grin2B* double mutants (herein notated as *grin2B*^*−/−*^) fish that are homozygous mutant for both the *grin2Ba* and *grin2Bb* alleles, thus lacking all GluN2B. *grin2B*^−/−^ larvae are viable, are recovered in Mendelian ratios, and show no gross morphological defects at 6 dpf (Fig. 2E). Surprisingly, the *grin2B*^−/−^ larvae also survive into adulthood and are fertile. This contrasts with mice lacking GluN2B, which display a suckling defect and die shortly after birth [23]. The extended survival of zebrafish lacking GluN2B enables study of the roles of this subunit throughout development.

### Zebrafish lacking GluN2B have impaired social behavior

**Fig. 3:**
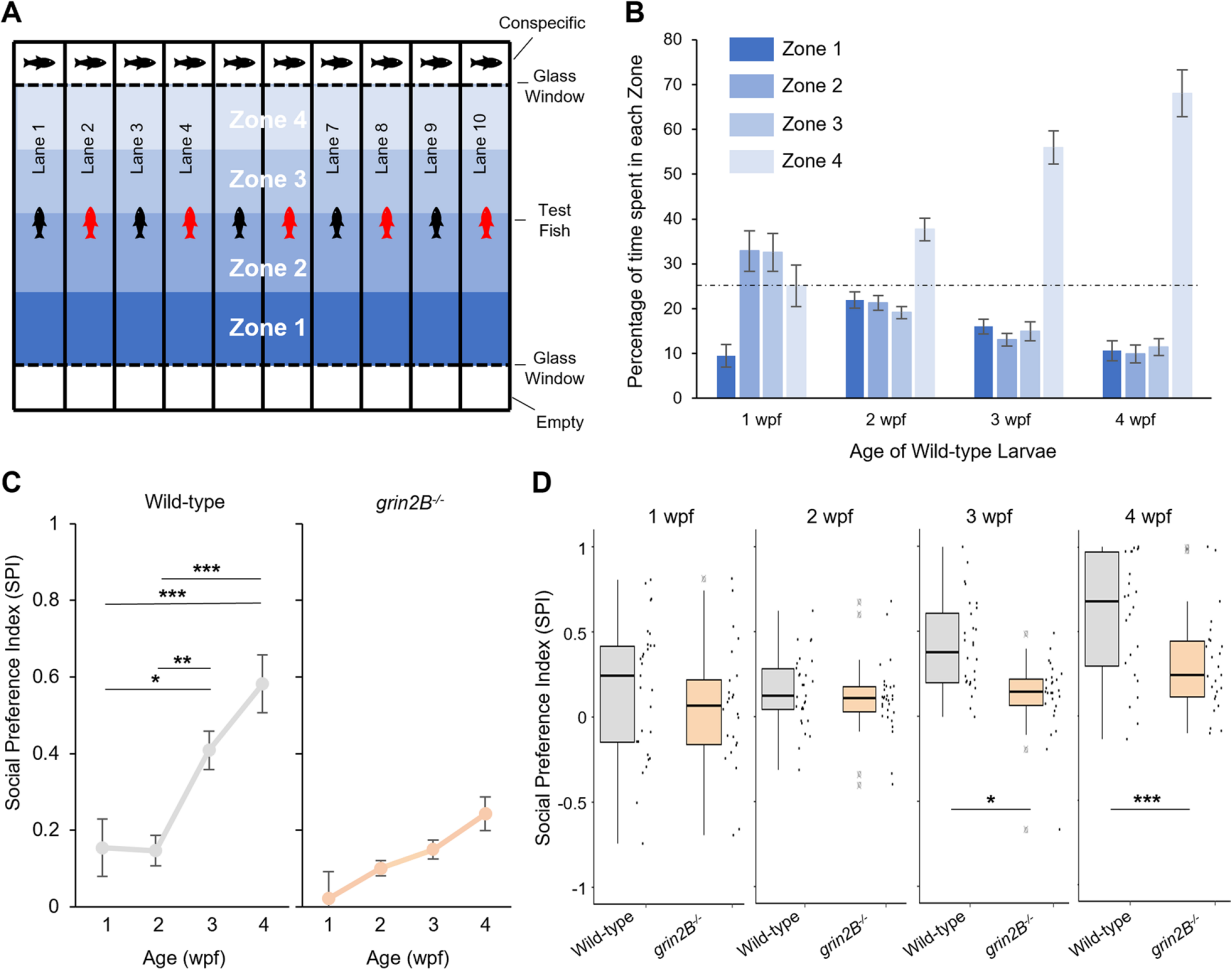
Fish lacking *grin2B* show a juvenile social deficit. **A** Schematic depiction of social behavior chamber. Each lane contains a test fish, either a wild-type control (depicted as a black fish) or a *grin2B*^−/−^ (depicted as a red fish). Individual fish can swim freely the length of the test lane. At the end of each lane is a clear glass window that is either empty (bottom row) or contains a wild-type age-matched conspecific (upper row) behind it. Each lane is subdivided into four equally sized zones, with Zone 4 the closest to the conspecific. **B** Bar graph (mean ± SEM) illustrating the time spent in each of the 4 zone subdivisions for either wild type at 1 (*n* = 29), 2 (*n* = 29), 3 (*n* = 28), or 4 (*n* = 24) wpf (weeks post fertilization). After allowing fish to briefly acclimate in the lane, fish were measured for 20 min. Dashed line is at 25%, the value of no preference for any zone. **C** Social Preference Index (SPI) (see “Materials and methods” section), a metric for social affinity. Values closer to 1 indicate a stronger social preference while 0 indicates no social preference. SPI was calculated at successive weeks for the experiment outlined in (**A**, **B**). Fish were assayed weekly from 1 to 4 wpf with experimental details in Additional file 1: Table S1. **D** Boxplots showing pairwise comparison of SPI for the same wild-type and *grin2B*^−/−^ fish at 1–4 wpf outlined in panel **C**. Individual dots represent SPI for individual fish with experimental details in Additional file 1: Table S1

Mutations in *GRIN2B* are associated with ASD in humans [46]. Because social deficits are a defining feature of ASD, we asked whether *grin2B*^−/−^ fish also display anomalous social behaviors. To assay social preference in zebrafish, we recorded activity of a freely swimming fish in a rectangular lane, where the short ends were separated by glass and contained either an age-matched wild-type conspecific or was empty (Fig. 3A). The apparatus consisted of 10 such lanes to allow assays of 10 fish simultaneously [33]. We divided each lane into 4 equal zones, with Zone 4 located adjacent to the conspecific.

Zebrafish develop a strong social preference to interact with conspecifics by 3 wpf (weeks post fertilization) [47]. Consistent with this finding, wild-type fish at 1 wpf spent approximately equal amounts of time in each zone, indicating no social preference, whereas fish at 3 or 4 wpf spent over half of their time in Zone 4, indicating a strong social preference (Fig. 3B). To quantify social preference of individual test fish, we used a social preference index (SPI) (Adapted from [33], see “Materials and methods” section). SPI values closer to + 1 indicate a strong social preference, those closer to − 1 indicate a social aversion, and those around 0 indicate no social preference. Consistent with the observations of zone preference (Fig. 3B), wild-type fish exhibit increased SPI at 3 and 4 wpf as compared to 1 and 2 wpf (Fig. 3C left panel). In contrast, *grin2B*^−/−^ fish do not show social preference over this same developmental period (Fig. 3C, right panel), although marginal non-significant increases were observed each week. When compared to age-matched wild-type controls, *grin2B*^−/−^ fish show significantly less social preference at both 3 and 4 wpf (Fig. 3D and Additional file 1: Table S1). This social preference deficit is not driven by the lack of a single *grin2B* paralogue, since the single *grin2Ba* and *grin2Bb* mutants show wild-type social behavior (Additional file 1: Fig. S2 and Additional file 1: Table S2).

Social preference in juvenile zebrafish results in a behavior coupling of social partners (visually driven coordinated swim bouts) in age-matched conspecifics [47]. Rodents show increased social preference to littermates [48]. Given this, two potential confounding factors for the *grin2B*^−/−^ social deficit in our assay are the combined inability for *grin2B*^−/−^ and wild-type fish to generate behavior coupling, and the possibility that wild-type zebrafish have increased social preference with fish from their same clutch. To test these possibilities, we used a modified social behavior paradigm in which all test fish were wild type, and the age-matched conspecifics were either *grin2B*^−/−^ or clutch-matched wild-type fish (Additional file 1: Fig. S3A). Wild-type test fish showed strong social preference to both wild-type and *grin2B*^−/−^ fish at 3 and 4 wpf, and there was no difference in the magnitude of social preference (Additional file 1: Fig. S3B and Additional file 1: Table S3). Hence, neither an inability to generate coupling, nor a clutch-preference, is the driving factor in the *grin2B*^*−/−*^ social deficit.

**Fig. 4:**
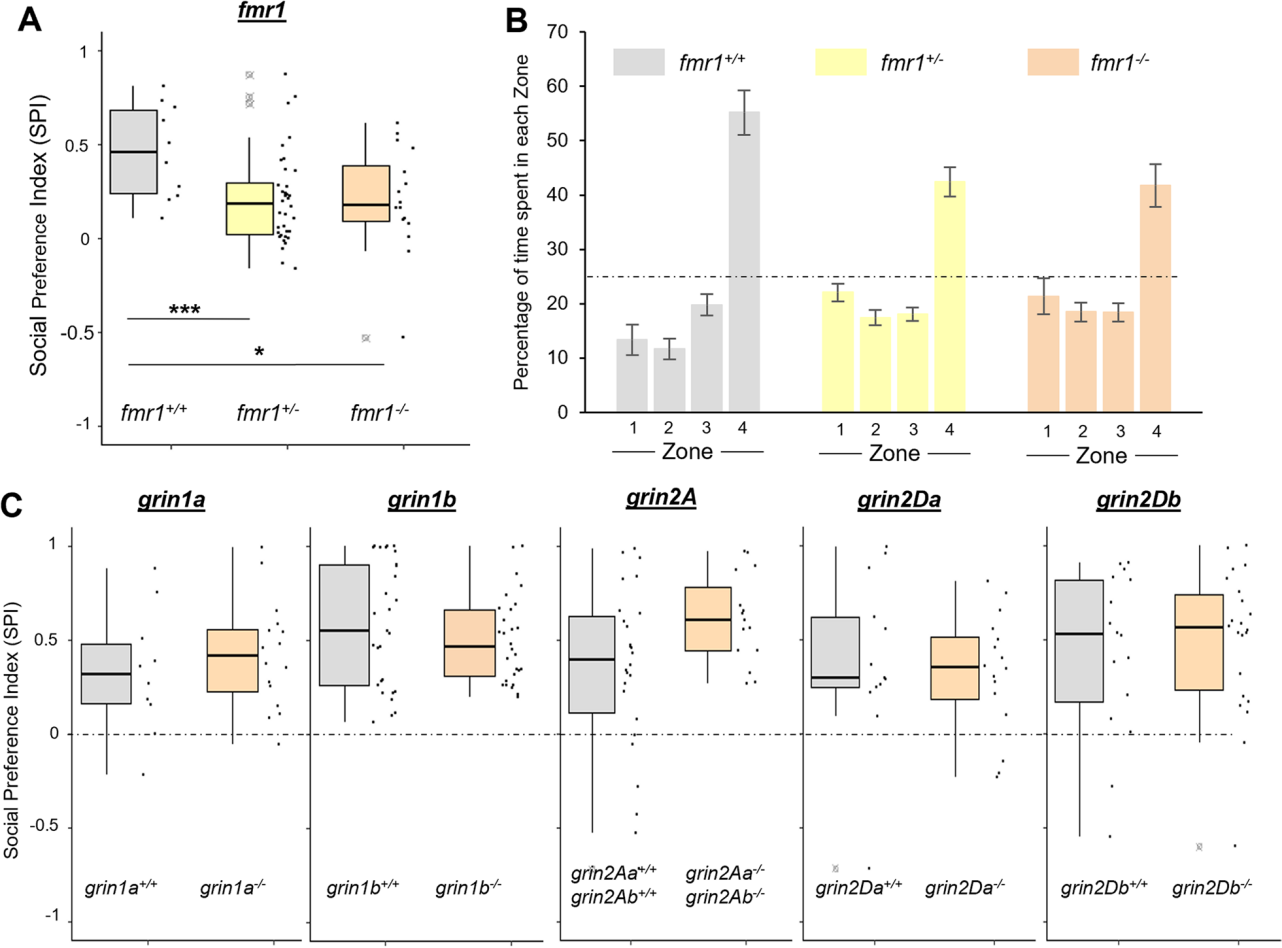
Zebrafish lacking *fmr1*, but not other NMDAR subunits, show a social preference deficit at 3 wpf. **A** SPI from a 3 wpf assay on fish lacking *fmr1*. Experimental details in Additional file 1: Table S4. **B** Bar graph (mean ± SEM) outlining mean time spent in each of the zone subdivisions for each *fmr1* genotype at 3 wpf. Dashed line is at 25%, the value of no preference for any zone. Zone 4 is the region closest to the conspecific. **C** SPI from 3 wpf assays of zebrafish lacking *grin1a, grin1b, grin2A, grin2Da,* and *grin2Db*. Dashed line is at 0, the value of no preference in SPI. Experimental details in Additional file 1: Table S4

*FMR1* dysfunction in humans is associated with Fragile X Syndrome, a neurodevelopmental disorder that exhibits social deficits [49]. We therefore tested zebrafish mutants lacking *fmr1* to further validate the capacity of our social behavior paradigm to detect social preference deficits. Like *grin2B*^−/−^ fish, zebrafish lacking *fmr1* showed a social deficit as compared to wild-type controls at 3 wpf characterized by a reduced SPI (Fig. 4A and Additional file 1: Table S4) and a reduced time spent in Zone 4 (Fig. 4B). Notably, these same deficits were observed in *fmr1* heterozygotes, indicating that the lack of only one *fmr1* allele is sufficient for the social preference deficit (Fig. 4A, B).

### Lack of GluN2A or mutations in additional NMDAR subunit paralogues do not produce social deficits

Non-specific pharmacological inhibition of all NMDARs blocks social preference [47], suggesting the *grin2B*^−/−^ social preference deficit might reflect a general phenomenon of reduced NMDAR activity and/or may be associated with any GluN2 subunit. To test these alternatives, we assayed social preference in fish lacking various NMDAR subunits (Fig. 4C and Additional file 1: Table S4). Initially, we tested fish lacking *grin1a* or *grin1b*, genes encoding the obligatory GluN1 subunit, but did not observe social deficits (Fig. 4C). To test the specificity of GluN2B-containing NMDARs in the development of social preference, we generated zebrafish knockouts in other NMDAR subunits (Additional file 1: Fig. S4). Zebrafish mutant for both *grin2Aa* and *grin2Ab*, and thus lacking all GluN2A, also did not show a social deficit (Fig. 4C). Additionally, no social deficit was observed in zebrafish lacking either *grin2Da* or *grin2Db*, genes encoding the GluN2D subunit. Together, these findings suggest that the lack of GluN2B specifically, and not other perturbations to NMDAR signaling, including a complete loss of GluN2A, is critical for the development of social behavior.

### Zebrafish lacking GluN2B exhibit numerous wild-type behaviors

The social deficit in *grin2B*^−/−^ fish could reflect a broad disruption of neurodevelopment and a lack of other basic behaviors and functions. To test this idea, we characterized additional behaviors.

**Fig. 5:**
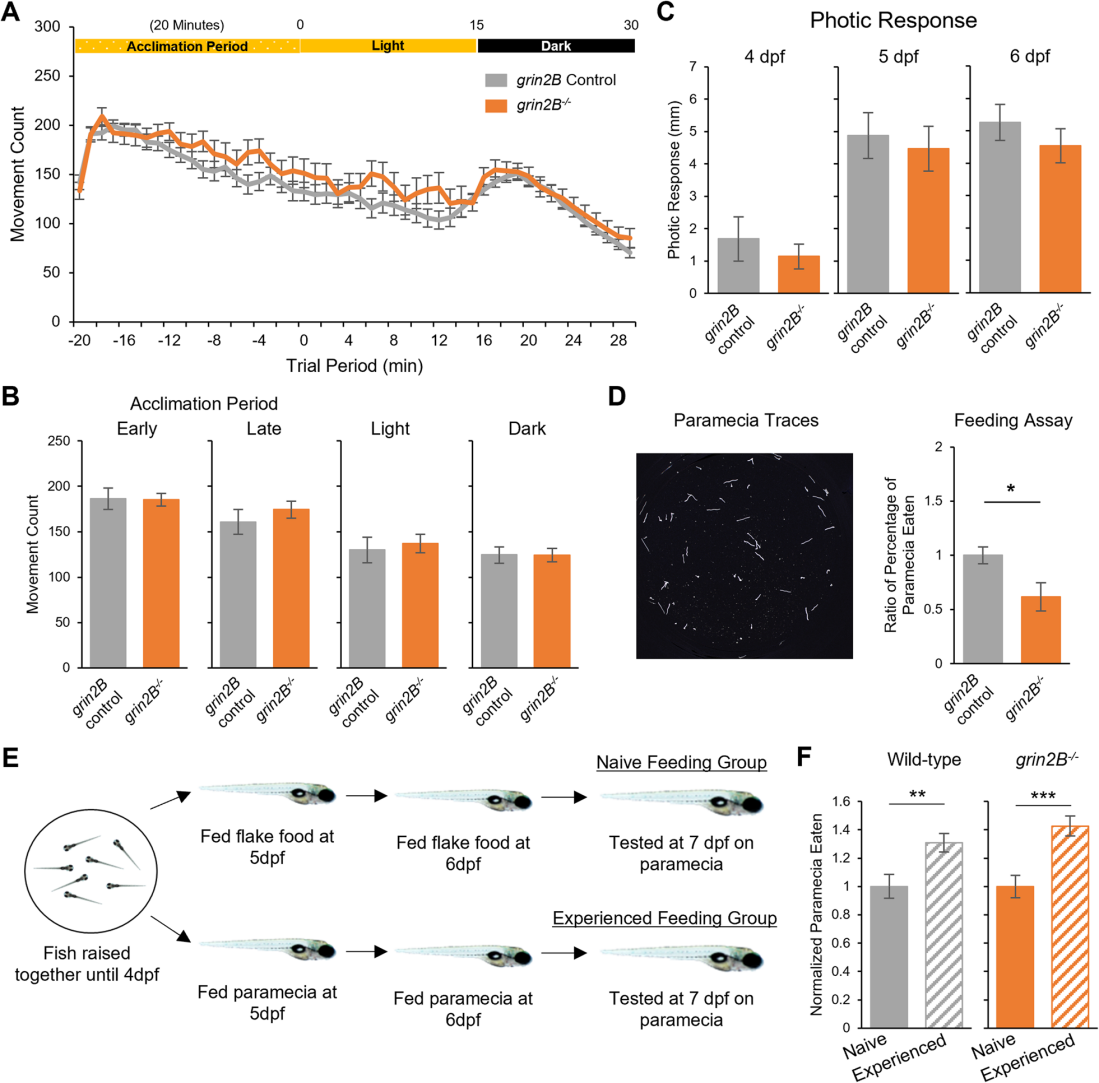
Zebrafish larvae lacking *grin2B* have wild-type spontaneous and photic-evoked swim behaviors and show the capacity to learn prey capture. **A** Spontaneous and photic-evoked larval swim behavior assay. Zebrafish larvae at 6 dpf acclimate to a behavior chamber for 20 min in the light and then are recorded for 15 min of spontaneous movement; after the removal of illumination, 15 min of behavior is measured in the dark. Line graph showing average movement count for a *grin2B* control (*n* = 70) and the *grin2B*^−/−^ fish (*n* = 30). Both control and mutant larvae exhibited the stereotypical visual motor response (VMR). **B** Bar graphs (mean ± SEM) of average movement count in each time period of the spontaneous and photic-evoked behavior outlined in (**A**). No differences were seen in any time period. **C** Zebrafish respond with a stereotyped startle response to the removal of illumination. Bar graphs showing the average distance traveled for a *grin2B* control (*n* = 19, 22, 22) and the *grin2B*^−/−^ fish (*n* = 27, 31, 31) for 4, 5, and 6 dpf, respectively, in the 1 s time period immediately following the removal of illumination. No difference was seen in the average response between groups at 4, 5, or 6 dpf. **D** Representative traces of paramecium movement used in feeding assay. Traces generated by analyzing 2.5 s of paramecia movement. Proportion of paramecia eaten over the trial period (mean ± SEM) normalized to control for**:** *grin2B* Control (*n* = 51) or *grin2B*^−/−^ (*n* = 20) fish at 7 dpf (*p = 0.011, *t* test). **E** Schematic of larval prey capture learning assay. **F** Proportion of paramecia eaten (mean ± SEM) in wild-type (left) (*n* = 35, 35. *p* = 0.005**, *t* test) and *grin2B*^−/−^ (*n* = 35, 32. *p* = 1.4e−4***, *t* test) for naïve (solid bars) and experienced (striped bars) fish, respectively. Proportion of paramecia eaten is normalized to the naïve feeding group

To assay the developing nervous system, we used a spontaneous and photic-evoked locomotion paradigm in which spontaneous movement is monitored in larvae at 6 dpf in 24-well plates for 35 min, followed by removal of illumination, which elicits a visual motor response, a stereotypical zebrafish behavior [50, 51]. Notably, there was no difference between wild type and *grin2B*^*−/−*^ fish in spontaneous movement in any period in the light, nor after the removal of illumination at 6 dpf (Fig. 5A, B) as well as at earlier developmental timepoints (4 and 5 dpf) (Additional file 1: Fig. S5). In alignment with these findings, *grin2B*^−/−^ fish showed no differences in total distance traveled during the social behavior paradigm at 3 or 4 weeks (Additional file 1: Fig. S6). Instantaneous response to a photic stimulus was measured by recording the distance traveled in the second after the removal of illumination. At 4, 5, and 6 dpf, *grin2B*^−/−^ larvae exhibited wild-type responses to this stimulus (Fig. 5C). Together, these findings indicate that *grin2B*^−/−^ larvae do not display broad behavioral deficits and can respond to external stimuli.

Some ASD patients also present with intellectual disabilities (ID) [5, 52]. To test whether *grin2B*^−/−^ larvae exhibit deficits in learning, we assayed them on a learned feeding assay. Prey capture is a complex behavior, necessitating visual and motor integration [53], and given experience, zebrafish show increases in both the number of attempted captures and successful captures of paramecia [54]. *grin2B*^−/−^ larvae are capable of capturing paramecia, but at a slightly diminished rate as compared to wild-type controls (Fig. 5D), a phenotype not seen in the *grin2Ba* or *grin2Bb* single mutants (Additional file 1: Fig. S7). To test whether the *grin2B*^−/−^ larvae were capable of learning, we assayed zebrafish that were either from a naïve feeding group (fed flake food at 5 and 6 dpf and assayed with paramecia at 7 dpf), or from an experienced feeding group (fed paramecia at 5 and 6 dpf and assayed with paramecia at 7 dpf) [54] (Fig. 5E). With experience, both wild-type and *grin2B*^−/−^ larvae significantly increase their successful paramecia captures and do so to a comparable extent (Fig. 5F). Hence, *grin2B*^*−/−*^ fish are capable of learning, at minimum within the scope of this paradigm, comparable to wild type.

### Compensatory changes in NMDAR subunit expression in ***grin2B***^***−/−***^

**Fig. 6:**
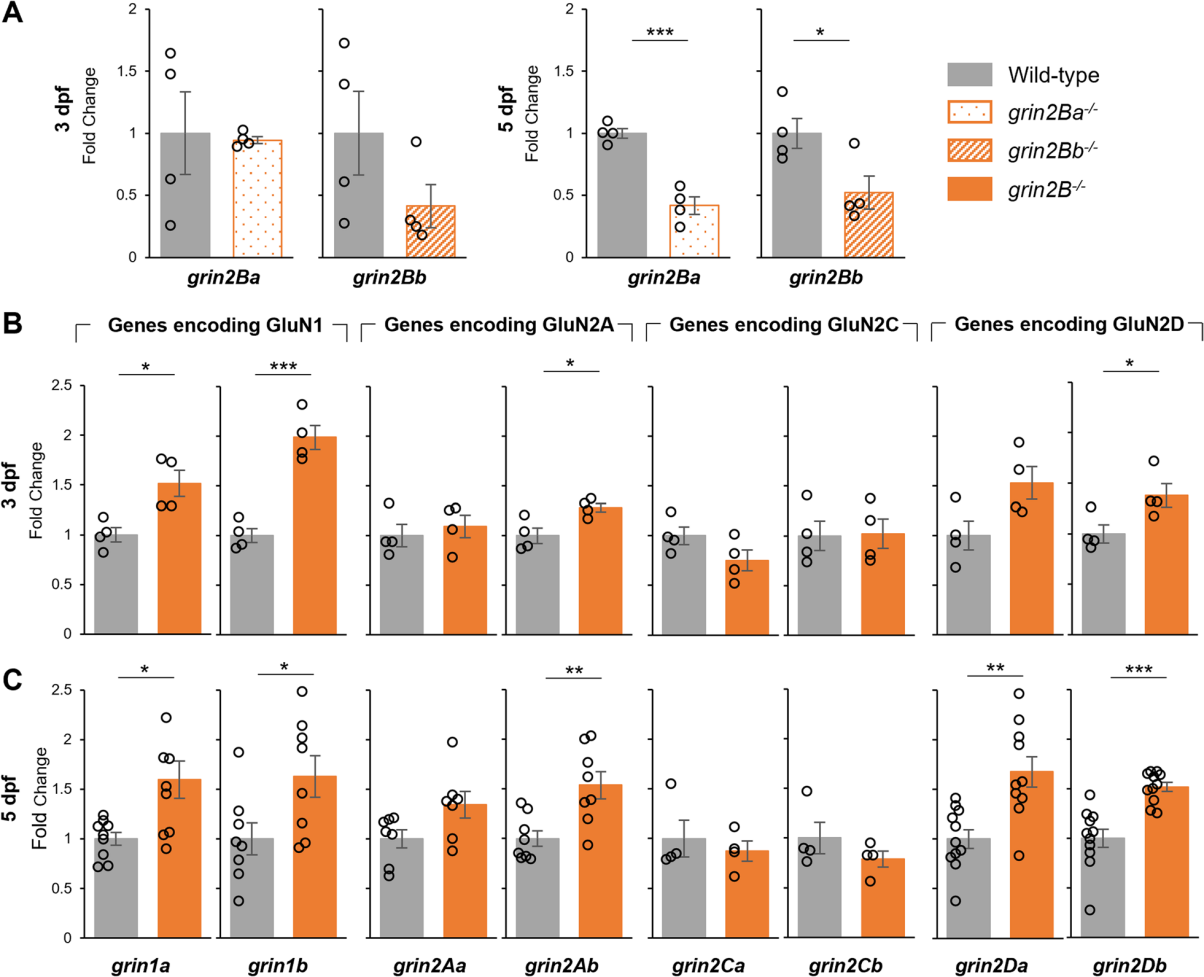
Nonsense-mediated decay and compensatory changes in NMDAR subunit expression in *grin2B*^*−/−*^. **A** Relative rt-qPCR expression levels at *grin2Ba* at 3 dpf (*n* = 4, 4) and 5 dpf (*n* = 4, 4; ***p = 3.5e−4) for wild-type controls and *grin2Ba* single mutants, respectively, and of *grin2Bb* at 3 dpf (*n* = 4, 4) and 5 dpf (*n* = 4, 4; *p = 0.037) for wild-type controls and *grin2Bb* single mutants, respectively. **B** Relative rt-qPCR expression levels at 3 dpf of *grin1a* (*n* = 4, 4; *p = 0.013), *grin1b* (*n* = 4, 4; ***p = 9.4e−4), *grin2Aa* (*n* = 4, 4), *grin2Ab* (*n* = 4, 4; *p = 0.026), *grinCa* (*n* = 4, 4), *grinCb* (*n* = 4, 4), *grinDa* (*n* = 4, 4), and *grinDb* (*n* = 4, 4; *p = 0.041) for wild type and *grin2B*^*−/−*^, respectively. **C** Relative rt-qPCR expression levels at 3 dpf of *grin1a* (*n* = 9, 9; *p = 0.013), *grin1b* (*n* = 8, 8; *p = 0.033), *grin2Aa* (*n* = 7, 7), *grin2Ab* (*n* = 8, 8; **p = 0.0061), *grinCa* (*n* = 4, 4), *grinCb* (*n* = 4, 4), *grinDa* (*n* = 11, 10; **p = 0.0018), and *grinDb* (*n* = 11, 11; ***p = 1.5e−4) for wild type and *grin2B*^*−/−*^, respectively

Nonsense-mediated decay (NMD) of mutant RNA can result in the transcriptional upregulation of similar genes [55]. To test whether NMD results from *grin2B* mutations, we assayed the levels of mutated *grin2Ba* and *grin2Bb* mRNA in their respective zebrafish mutants at 3 and 5 dpf (Fig. 6A). At 3 dpf, no statistically significant changes were observed, whereas at 5 dpf, a significant decrease in mutant mRNA was reported in both paralogues. Together, this suggests progressive NMD in the *grin2B*^−/−^ larvae.

NMDAR subunits share similar structural domains and may thus be targets of transcriptional adaptation as the result of NMD. To test the possible upregulation of corresponding NMDAR subunits, we assayed the levels of the genes encoding GluN1, GluN2A, GluN2C and GluN2D. Upregulation was observed in *grin1a, grin1b, grin2Ab*, and *grin2Db* at 3 dpf (Fig. 6B). No change in transcript levels was reported in either *grin2C* paralogue. If NMD is the driving factor of these transcriptional changes, we would predict similar but potentially increased changes at 5 dpf, where the level of NMD in the *grin2B* mRNA transcripts was higher. Indeed, all the transcriptional upregulations reported at 3 dpf, as well as increases in *grin2Da,* were observed at 5 dpf, often to a greater magnitude (Fig. 6C). Together, these findings indicate a transcriptional upregulation of specific NMDAR subunits, potentially driven by NMD and transcriptional adaptation.

### Whole-brain imaging of ***grin2B***^***−/−***^ fish reveals reduced inhibitory neuron signal in the subpallium but no gross anatomical changes

Various changes in brain architecture and neuron populations have been described in ASD patients, including alterations to *E*/*I* balances often due to reductions in inhibitory neuron populations [56–58]. To test if *grin2B*^−/−^ fish had any such changes, we performed whole-brain confocal scans of 6 dpf larvae. *grin2B*^−/−^ larvae and controls containing transgenic lines labeling excitatory (*vglut:DsRed*) and inhibitory (*dlx6a-1.4kbdlx5a/dlx6a:GFP)* populations were mapped onto a reference using the Zebrafish Brain Browser [38].

**Fig. 7:**
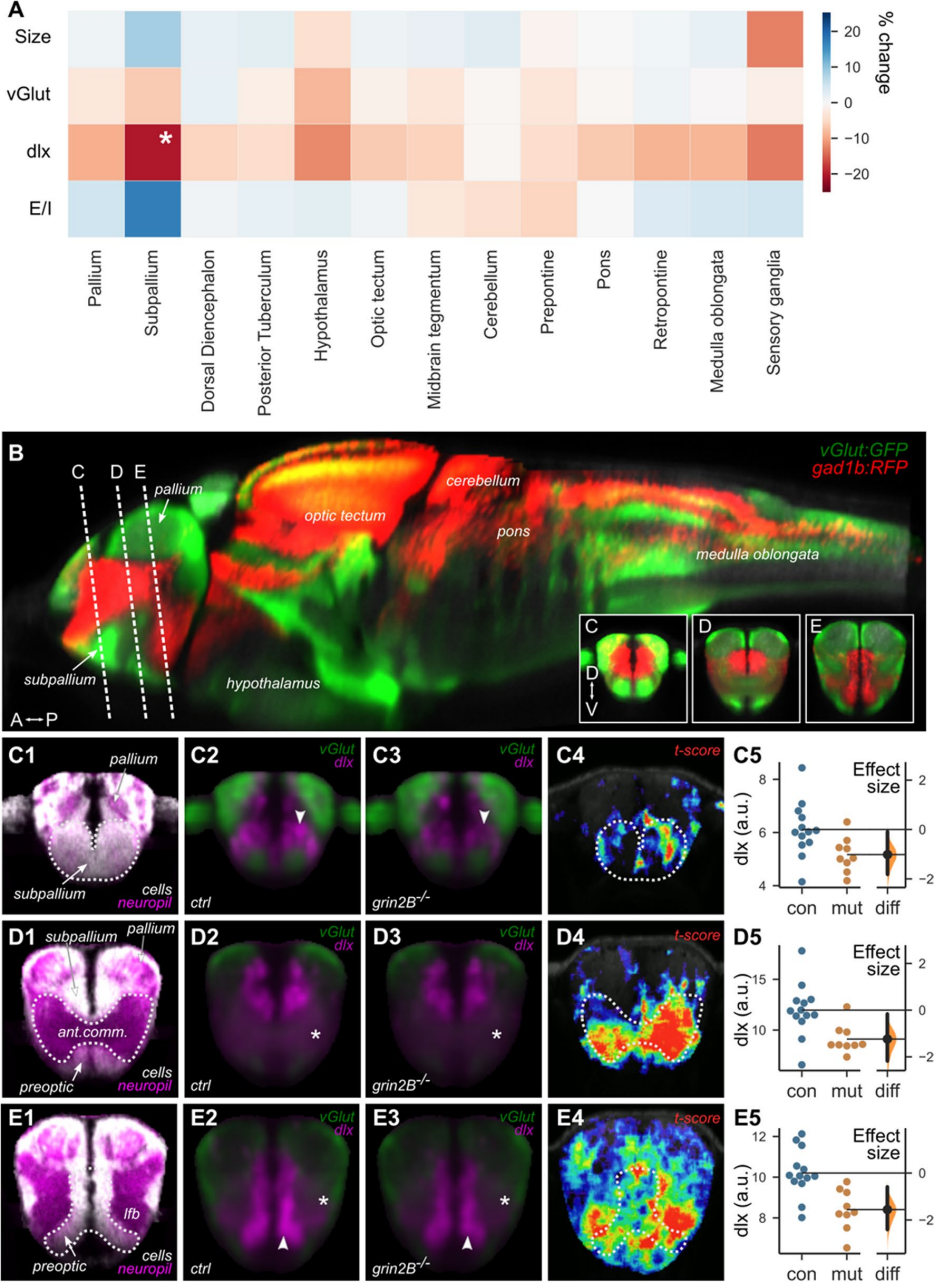
Whole-brain imaging of zebrafish lacking GluN2B reveal a reduced inhibitory neuron signal in the subpallium. **A** Heat map of the ratio of size, transgenic expression of excitatory (vGlut) and inhibitory (dlx) marker, and calculated excitatory/inhibitory balance (*E*/*I*) for *grin2B* control (*n* = 13) and *grin2B*^−/−^ (*n* = 9) larvae. Values are shown as the percent increase in the signal *grin2B*^−/−^ larvae compared to *grin2B* control. (*p = 0.022, *independent-samples t test*). **B** Reconstructed sagittal section from the Zebrafish Brain Browser (ZBB) showing the distribution of glutamatergic (green) and GABAergic (red) neurons in the larval brain. Planes through which coronal sections were reconstructed for panels **C**–**E** are indicated. Anterior (A), posterior (P), dorsal (D), ventral (V). In Panel **B**, C1, D1, and E1 were generated using the Zebrafish Brain Browser. Subpanels 2–4 from (**C**–**E**) were generated using data acquired from dlx:GFP larvae. **C**–**E** Coronal sections through parts of the subpallium, including the anterior cell-rich domain (**C**), anterior commissure (**D**), and preoptic region (**E**). Subpanel 1: distribution of cell-rich (white) and neuropil-rich (magenta) zones (ZBB). Subpanels 2–3: mean confocal signal in *grin2B* control and *grin2B*^−/−^ larvae, for excitatory (Tg(vglut:DsRed); green) and inhibitory (Tg(dlx6a-1.4kbdlx5a/dlx6a:GFP); magenta) neurons. Subpanel 4: spatial locations of voxel-level normalized differences (t-score) between the inhibitory signal in *grin2B* control and *grin2B*^−/−^ larvae. Subpanel 5: Estimation plots showing mean intensity of the inhibitory signal per fish, for regions encompassing the anterior subpallium (**C**, *p* = 0.015), anterior commissure (**D**, *p* = 0.0038), and preoptic region (**E**, *p* = 0.0011) for *grin2B* control (blue) and *grin2B*^−/−^ (orange) larvae. Right axes show effect sizes for differences

*grin2B*^−/−^ larvae (*n* = 9) had no significant changes in gross brain size [106% the size of *grin2B* controls (*n* = 13)], or overall signal from excitatory or inhibitory neurons (99 and 92% the signal, respectively, of controls). Upon subdividing the brain into major divisions, however, we found a significant 20.2% decrease in the mean inhibitory neuron signal in the subpallium (Fig. 7A). To examine this more closely, we mapped voxel-level differences in inhibitory neuron signal to coronal sections through different parts of the subpallium, reconstructed from confocal images (Fig. 7B). As for many other zebrafish brain regions, the subpallium has cell-rich and neuropil-rich zones. A significant reduction in the inhibitory neuron signal was present in the cell-rich anterior subpallial domain (Fig. 7C). However, greater reductions were seen in subpallial areas posterior to this region, encompassing the anterior commissure (Fig. 7D) and the lateral forebrain bundle, which is adjacent to the preoptic region (Fig. 7E). These findings highlight inhibitory neuron populations in specific subpallial brain regions as a key alteration caused by developmental lack of GluN2B.

## Discussion

*GRIN2B* is a high-confidence ASD-associated gene in ASD [8, 11, 19]. How it contributes to clinical phenotypes, however, is poorly understood. In addition to well-studied functions for NMDARs in synaptic transmission at mature excitatory synapses, NMDARs also play key roles in development [13]. NMDARs regulate neurogenesis [20], cell migration [59], and neuronal differentiation [22]. Here, we establish zebrafish as an in vivo model to study GluN2B in development and dysfunction. Unlike mouse knockouts for *GRIN2B,* which fail to suckle and die shortly after birth [23], zebrafish lacking GluN2B (*grin2B*^−/−^ fish) survive into adulthood and are fertile (Fig. 2). Notably, many aspects of these *grin2B*^−/−^ fish appear normal including movement (Fig. 5A–C) and learning (Fig. 5E, F) which may be driven by the compensational expression of other NMDAR genes (Fig. 6). Nevertheless, one behavior—a juvenile social preference—is deficient in *grin2B*^−/−^ fish (Fig. 3). Social deficits are common in ASD patients, and the specificity of this behavioral phenotype in this model is a powerful tool to study *grin2B* in brain dysfunction. Altered excitatory/inhibitory (*E*/*I*) balance, often caused by reductions in inhibitory neuron populations, is central to ASD pathogenesis [58, 60]. We did not see dramatic changes in *E*/*I* balance when globally observing the brain in *grin2B*^−/−^ fish (Fig. 7). However, we observed a significant reduction in an inhibitory neuron marker in the subpallium, a region that will later mature into putative homologues of amygdaloid, septal, and striatal nuclei, all structures previously implicated in ASD [28, 29, 61, 62] (Additional file 1: Table S5).

Due to an ancient genome duplication, zebrafish have two *grin2B* paralogues (*grin2Ba* and *grin2Bb*) [63]. Proteins generated from both paralogues share considerable sequence homology with each other and human GluN2B (hGluN2B), only diverging in sequence in the amino-terminal (ATD) and carboxy-terminal (CTD) domains (Fig. 1). The CTD is the region of greatest variance in sequence and length among NMDAR subunits of the same species and is involved in intracellular signaling [15]. Despite this, zebrafish GluN2B proteins interact with various conserved intracellular components, including Ca^2+^/calmodulin-dependent protein kinase (CAMKII) [64] and ERK [65]. As suggested by the conservation in sequence in ligand-binding, transmembrane, and pore-forming domains, zGluN2Bb formed functional heterotetramers with hGluN1 in a heterologous expression system (Fig. 1C). These cross-species NMDARs showed glutamate-gated inward current and had prolonged deactivation, a hallmark of GluN2B-containing NMDARs (Fig. 1E). These results demonstrate a high degree of functional similarity between human and zebrafish GluN2B proteins.

There are multiple lines of evidence supporting the claim that there is no residual GluN2B function in our *grin2B*^*−/−*^ zebrafish. Firstly, we generated our CRISPR-Cas9 mutations directly before or in the critical M3 pore-lining helix (Fig. 2A–C). Due to the importance of this highly conserved region, any alternative-splicing event that restored the reading frame of the protein would still render a non-functional channel. Additionally, no wild-type mRNA was detected for either *grin2Ba* or *grin2Bb* in our *grin2B*^*−/−*^ zebrafish (Fig. 2D). The observation of NMD and potential transcriptional adaptation also supports that the mutant mRNA is targeted for degradation (Fig. 6).

Zebrafish are social creatures. Non-specific block of all NMDARs with MK-801 decreases social preference in juveniles [47, 66] and adults [66, 67]. Our findings refine this pharmacological work by implicating GluN2B, rather than general NMDAR dysfunction, in the development of social preference (Fig. 3D). Additionally, acute pharmacological block precludes the understanding of any developmental roles of NMDARs. It is possible that MK-801-driven disruptions in movement control may also contribute to observed social deficits in these assays [34, 47, 66]. Our findings suggest that developmental alterations in specific brain regions, rather than alterations in movement control (Fig. 5), are likely generating these phenotypes.

Disease-associated missense mutations in *GRIN2B* are more likely to result in neurodevelopmental disorders, such as ASD, as compared to other subunits, highlighting the significant developmental requirements for GluN2B [68]. Interestingly, although not as numerous as in GluN2B, rare variants associated with ASD are also found in GluN1, GluN2A, GluN2C, and GluN2D [69]. Zebrafish lacking other NMDAR subunit paralogues (*grin1a, grin1b, grin2Aa/b, grin2Da, or grin2Db*) did not result in a social deficit at 3 wpf. Only through testing *grin2C* and *grin2D* double mutants (as done here with *grin2A*) can we definitively rule out the involvement of these subunits. However, given the lack of social behavioral phenotype in fish lacking the widely expressed GluN2A subunit, mutations in other subunits may confer some effects through association with GluN2B, possibly in triheteromeric receptors.

Behavior and cellular phenotypes of *fmr1* mutant fish have been previously studied [70–72]. Interestingly, our findings on *fmr1* social deficits (Fig. 4A, B) differ from previous studies which report *fmr1* mutant zebrafish developing social preference earlier and more robustly than wild type [73]. These studies use a different social behavior paradigm that may be more influenced by well-documented hyperactivity in *fmr1* mutant zebrafish [73, 74].

Zebrafish lacking GluN2B displayed wild-type spontaneous swimming, visual motor response, photic startle responses and could capture paramecia, albeit at a slightly diminished capacity (Fig. 5). Further, these fish were capable of learning within the prey capture paradigm (Fig. 5F). One possibility is that synaptic plasticity mediated by GluN2B-containing NMDARs is not essential to learning the prey capture task. Alternatively, the action of other NMDARs, such as those containing GluN2A or GluN2D, can compensate for the lack of GluN2B. Indeed, in rodents, increase in levels of GluN1 and GluN2A was observed during memory consolidation, while GluN2B levels remained unaltered [75].

NMD of mutant RNA can result in transcriptional adaptation [55]. Compensatory increases in other NMDAR subunits were observed in our assays (Fig. 6) and may contribute to both the viability of the *grin2B*^*−/−*^ fish into adulthood, as well as their wild-type spontaneous locomotion, photic-evoked responses, and capacity to learn in prey capture assays. These findings, however, further highlight the specificity of GluN2B in the development of social behavior, as upregulation of other NMDAR subunits is not sufficient to rescue this phenotype.

Whole-brain scans of zebrafish larvae lacking GluN2B and containing transgenic lines labeling excitatory and inhibitory populations outlined no structural or whole-brain changes in neuronal populations. Similar whole-brain larval confocal scans have been done on valproic acid (VPA)-treated fish [40]. VPA exposure in zebrafish also causes social deficits [76, 77] and is associated with increased risk of ASD in humans [78, 79]. Unsurprisingly, due to the broad disruption caused by global VPA treatment, gross structural and compositional changes were reported in many regions [40]. Given the shared social phenotype of VPA-treated and *grin2B*^*−/−*^ fish, our model offers a more refined view of the potential regions involved in establishing this phenotype. In particular, we observed a significant reduction in the intensity of the marker labeling inhibitory neurons in the subpallium of 6 dpf larvae. Changes were apparent in a cell-rich anterior domain and in neuropil-rich regions surrounding the preoptic area.

Nuclei of the precommissural subpallium are outlined on a schematic coronal section of the telencephalon (Additional file 1: Fig. S8). At this stage, major regions of the forebrain are not yet mature; however, neurons derived from the subpallium later contribute to putative zebrafish homologues of the amygdala, septum, and striatum [80–82] (Additional file 1: Table S5). While precise homologies of specific zebrafish nuclei remain unresolved, there are indisputably domains of the zebrafish ventral telencephalon (subpallium) that molecularly and functionally represent these structures. Our data suggest that changes in these regions may underlie the abnormal social behavior observed in 3–4 weeks old *grin2B*^*−/−*^ fish. Abnormalities in amygdalar, septal, and striatal development and function have been implicated in ASD and are thought to contribute to the social impairment that characterizes this disorder [28, 29, 61, 62]. Given the autonomic dysfunction and sensory processing atypia associated with ASD, it is unsurprising that we also uncovered substantial differences in inhibitory neuron signal in additional anatomical areas related to these functions [83–85]. These findings and their associations warrant further investigation.

We establish zebrafish as a clinically relevant model for investigating developmental roles of GluN2B. The viability, homology, and ability to assay social preference together facilitate the study of GluN2B in development and ASD etiology. Given the myriad roles of NMDAR in development, further study into the specific mechanisms by which GluN2B perturbation gives rise to the ASD phenotype are necessary. These should include effects on neurogenesis, connectivity, and synaptic alterations with a specific focus on the nuclei of the subpallium. In addition to knockout models, the advent of precise gene-editing in zebrafish [86], coupled with the high homology of GluN2B in zebrafish, facilitates the future study of ASD-associated GluN2B missense mutations in zebrafish.

## Supplementary Information

**Additional file 1**. **Supplementary Figures 1–8 and Supplementary Tables 1–5**: **Figure S1**. Alignments of hGluN2B, zGluN2Ba and zGluN2Bb. **Table S1**. Social behavior for *grin2B*^-/-^ fish. **Figure S2**. *grin2B* single mutants do not show a juvenile social deficit. **Table S2**. Social behavior for *grin2B* single mutant fish. **Figure S3**. Wild-type fish show no preference for social interactions with wild-type when compared to *grin2B*^-/-^. **Table S3**. Social behavior with *grin2B*^-/-^ as conspecific. **Table S4**. Social behavior for an ASD-associated gene and other NMDAR subunits. **Figure S4**. Generation of loss-of-function lesions in *grin2A* and* grin2D* subunit paralogues. **Figure S5**. *grin2B*^-/-^ fish show wild-type spontaneous and photic evoked responses throughout early larval stages. **Figure S6**. *grin2B*^-/-^ have normal swim behavior at 3 or 4wpf. **Figure S7**. *grin2B* single mutants do not show a larval feeding deficit. **Figure S8**. Zebrafish subpallium schematic. **Table S5**. Proposed mammalian homologies for zebrafish subpallial nuclei.

## Data Availability

Please contact corresponding author for data requests.

## References

[1] Zeidan J, Fombonne E, Scorah J, Ibrahim A, Durkin MS, Saxena S (2022). Global prevalence of autism: a systematic review update. Autism Res.

[2] Lai MC, Lombardo MV, Baron-Cohen S (2014). Autism. Lancet.

[3] Roman-Urrestarazu A, van Kessel R, Allison C, Matthews FE, Brayne C, Baron-Cohen S (2021). Association of race/ethnicity and social disadvantage with autism prevalence in 7 million school children in England. JAMA Pediatr.

[4] American Psychiatric Association (2013). Diagnostic and statistical manual of mental disorders.

[5] Baio J, Wiggins L, Christensen DL, Maenner MJ, Daniels J, Warren Z (2018). Prevalence of autism spectrum disorder among children aged 8 years—autism and developmental disabilities monitoring network, 11 sites, United States, 2014. MMWR Surveill Summ.

[6] Fombonne E (2009). A wrinkle in time: from early signs to a diagnosis of autism. J Am Acad Child Adolesc Psychiatry.

[7] Iossifov I, Ronemus M, Levy D, Wang Z, Hakker I, Rosenbaum J (2012). De novo gene disruptions in children on the autistic spectrum. Neuron.

[8] O'Roak BJ, Vives L, Fu W, Egertson JD, Stanaway IB, Phelps IG (2012). Multiplex targeted sequencing identifies recurrently mutated genes in autism spectrum disorders. Science.

[9] De Rubeis S, Buxbaum JD (2015). Recent advances in the genetics of autism spectrum disorder. Curr Neurol Neurosci Rep.

[10] Stessman HA, Xiong B, Coe BP, Wang T, Hoekzema K, Fenckova M (2017). Targeted sequencing identifies 91 neurodevelopmental-disorder risk genes with autism and developmental-disability biases. Nat Genet.

[11] Satterstrom FK, Kosmicki JA, Wang J, Breen MS, De Rubeis S, An JY (2020). Large-scale exome sequencing study implicates both developmental and functional changes in the neurobiology of autism. Cell.

[12] Hansen KB, Wollmuth LP, Bowie D, Furukawa H, Menniti FS, Sobolevsky AI (2021). Structure, function, and pharmacology of glutamate receptor ion channels. Pharmacol Rev.

[13] Chakraborty A, Murphy S, Coleman N (2017). The role of NMDA receptors in neural stem cell proliferation and differentiation. Stem Cells Dev.

[14] Hunt DL, Castillo PE (2012). Synaptic plasticity of NMDA receptors: mechanisms and functional implications. Curr Opin Neurobiol.

[15] Paoletti P, Bellone C, Zhou Q (2013). NMDA receptor subunit diversity: impact on receptor properties, synaptic plasticity and disease. Nat Rev Neurosci.

[16] Yuan H, Low CM, Moody OA, Jenkins A, Traynelis SF (2015). Ionotropic GABA and glutamate receptor mutations and human neurologic diseases. Mol Pharmacol.

[17] Hardingham GE, Do KQ (2016). Linking early-life NMDAR hypofunction and oxidative stress in schizophrenia pathogenesis. Nat Rev Neurosci.

[18] Geisheker MR, Heymann G, Wang T, Coe BP, Turner TN, Stessman HAF (2017). Hotspots of missense mutation identify neurodevelopmental disorder genes and functional domains. Nat Neurosci.

[19] O'Roak BJ, Vives L, Girirajan S, Karakoc E, Krumm N, Coe BP (2012). Sporadic autism exomes reveal a highly interconnected protein network of de novo mutations. Nature.

[20] Nacher J, McEwen BS (2006). The role of *N*-methyl-D-asparate receptors in neurogenesis. Hippocampus.

[21] Bagasrawala I, Memi F, Radonjić VN, Zecevic N (2017). *N*-Methyl d-aspartate receptor expression patterns in the human fetal cerebral cortex. Cereb Cortex.

[22] Bell S, Maussion G, Jefri M, Peng H, Theroux JF, Silveira H (2018). Disruption of GRIN2B impairs differentiation in human neurons. Stem Cell Rep.

[23] Kutsuwada T, Sakimura K, Manabe T, Takayama C, Katakura N, Kushiya E (1996). Impairment of suckling response, trigeminal neuronal pattern formation, and hippocampal LTD in NMDA receptor epsilon 2 subunit mutant mice. Neuron.

[24] Rea V, Van Raay TJ (2020). Using zebrafish to model autism spectrum disorder: a comparison of ASD risk genes between zebrafish and their mammalian counterparts. Front Mol Neurosci.

[25] Fedele L, Newcombe J, Topf M, Gibb A, Harvey RJ, Smart TG (2018). Disease-associated missense mutations in GluN2B subunit alter NMDA receptor ligand binding and ion channel properties. Nat Commun.

[26] Fontana BD, Muller TE, Cleal M, de Abreu MS, Norton WHJ, Demin KA (2022). Using zebrafish (*Danio rerio*) models to understand the critical role of social interactions in mental health and wellbeing. Prog Neurobiol.

[27] Cox JA, Kucenas S, Voigt MM (2005). Molecular characterization and embryonic expression of the family of *N*-methyl-d-aspartate receptor subunit genes in the zebrafish. Dev Dyn.

[28] Fuccillo MV (2016). Striatal circuits as a common node for autism pathophysiology. Front Neurosci.

[29] Janouschek H, Chase HW, Sharkey RJ, Peterson ZJ, Camilleri JA, Abel T (2021). The functional neural architecture of dysfunctional reward processing in autism. Neuroimage Clin.

[30] Yelshansky MV, Sobolevsky AI, Jatzke C, Wollmuth LP (2004). Block of AMPA receptor desensitization by a point mutation outside the ligand-binding domain. J Neurosci.

[31] Alsaloum M, Kazi R, Gan Q, Amin J, Wollmuth LP (2016). A molecular determinant of subtype-specific desensitization in ionotropic glutamate receptors. J Neurosci.

[32] Amin JB, Salussolia CL, Chan K, Regan MC, Dai J, Zhou HX (2017). Divergent roles of a peripheral transmembrane segment in AMPA and NMDA receptors. J Gen Physiol.

[33] Geng Y, Zhang T, Godar SC, Pluimer BR, Harrison DL, Nath AK (2021). Top2a promotes the development of social behavior via PRC2 and H3K27me3. bioRxiv.

[34] Zoodsma JD, Chan K, Bhandiwad AA, Golann D, Liu G, Syed S (2020). A model to study NMDA receptors in early nervous system development. J Neurosci.

[35] Ghanem N, Jarinova O, Amores A, Long Q, Hatch G, Park BK (2003). Regulatory roles of conserved intergenic domains in vertebrate Dlx bigene clusters. Genome Res.

[36] Kinkhabwala A, Riley M, Koyama M, Monen J, Satou C, Kimura Y (2011). A structural and functional ground plan for neurons in the hindbrain of zebrafish. Proc Natl Acad Sci USA.

[37] Preibisch S, Saalfeld S, Tomancak P (2009). Globally optimal stitching of tiled 3D microscopic image acquisitions. Bioinformatics.

[38] Tabor KM, Marquart GD, Hurt C, Smith TS, Geoca AK, Bhandiwad AA (2019). Brain-wide cellular resolution imaging of Cre transgenic zebrafish lines for functional circuit-mapping. Elife.

[39] Marquart GD, Tabor KM, Brown M, Strykowski JL, Varshney GK, LaFave MC (2015). A 3D searchable database of transgenic zebrafish Gal4 and Cre lines for functional neuroanatomy studies. Front Neural Circuits.

[40] Gupta T, Marquart GD, Horstick EJ, Tabor KM, Pajevic S, Burgess HA (2018). Morphometric analysis and neuroanatomical mapping of the zebrafish brain. Methods.

[41] Ho J, Tumkaya T, Aryal S, Choi H, Claridge-Chang A (2019). Moving beyond P values: data analysis with estimation graphics. Nat Methods.

[42] Amin JB, Leng X, Gochman A, Zhou HX, Wollmuth LP (2018). A conserved glycine harboring disease-associated mutations permits NMDA receptor slow deactivation and high Ca(2+) permeability. Nat Commun.

[43] Chang N, Sun C, Gao L, Zhu D, Xu X, Zhu X (2013). Genome editing with RNA-guided Cas9 nuclease in zebrafish embryos. Cell Res.

[44] Hwang WY, Fu Y, Reyon D, Maeder ML, Tsai SQ, Sander JD (2013). Efficient genome editing in zebrafish using a CRISPR-Cas system. Nat Biotechnol.

[45] Wollmuth LP, Sobolevsky AI (2004). Structure and gating of the glutamate receptor ion channel. Trends Neurosci.

[46] Hu C, Chen W, Myers SJ, Yuan H, Traynelis SF (2016). Human GRIN2B variants in neurodevelopmental disorders. J Pharmacol Sci.

[47] Dreosti E, Lopes G, Kampff AR, Wilson SW (2015). Development of social behavior in young zebrafish. Front Neural Circuits.

[48] Clemens AM, Brecht M (2021). Neural representations of kinship. Curr Opin Neurobiol.

[49] Brodkin ES (2008). Social behavior phenotypes in fragile X syndrome, autism, and the Fmr1 knockout mouse: theoretical comment on McNaughton et al. (2008). Behav Neurosci.

[50] Burgess HA, Granato M (2007). Modulation of locomotor activity in larval zebrafish during light adaptation. J Exp Biol.

[51] Emran F, Rihel J, Dowling JE (2008). A behavioral assay to measure responsiveness of zebrafish to changes in light intensities. J Vis Exp.

[52] Al-Beltagi M (2021). Autism medical comorbidities. World J Clin Pediatr.

[53] Semmelhack JL, Donovan JC, Thiele TR, Kuehn E, Laurell E, Baier H (2014). A dedicated visual pathway for prey detection in larval zebrafish. Elife.

[54] Oldfield CS, Grossrubatscher I, Chavez M, Hoagland A, Huth AR, Carroll EC (2020). Experience, circuit dynamics, and forebrain recruitment in larval zebrafish prey capture. Elife.

[55] El-Brolosy MA, Kontarakis Z, Rossi A, Kuenne C, Gunther S, Fukuda N (2019). Genetic compensation triggered by mutant mRNA degradation. Nature.

[56] Courchesne E, Pramparo T, Gazestani VH, Lombardo MV, Pierce K, Lewis NE (2019). The ASD living biology: from cell proliferation to clinical phenotype. Mol Psychiatry.

[57] Uzunova G, Pallanti S, Hollander E (2016). Excitatory/inhibitory imbalance in autism spectrum disorders: implications for interventions and therapeutics. World J Biol Psychiatry.

[58] Contractor A, Ethell IM, Portera-Cailliau C (2021). Cortical interneurons in autism. Nat Neurosci.

[59] Namba T, Ming GL, Song H, Waga C, Enomoto A, Kaibuchi K (2011). NMDA receptor regulates migration of newly generated neurons in the adult hippocampus via Disrupted-In-Schizophrenia 1 (DISC1). J Neurochem.

[60] Bruining H, Hardstone R, Juarez-Martinez EL, Sprengers J, Avramiea AE, Simpraga S (2020). Measurement of excitation–inhibition ratio in autism spectrum disorder using critical brain dynamics. Sci Rep.

[61] Horiai M, Otsuka A, Hidema S, Hiraoka Y, Hayashi R, Miyazaki S (2020). Targeting oxytocin receptor (Oxtr)-expressing neurons in the lateral septum to restore social novelty in autism spectrum disorder mouse models. Sci Rep.

[62] Shen MD, Swanson MR, Wolff JJ, Elison JT, Girault JB, Kim SH (2022). Subcortical brain development in autism and fragile X syndrome: evidence for dynamic, age- and disorder-specific trajectories in infancy. Am J Psychiatry.

[63] Amores A, Force A, Yan YL, Joly L, Amemiya C, Fritz A (1998). Zebrafish hox clusters and vertebrate genome evolution. Science.

[64] Roy B, Ferdous J, Ali DW (2015). NMDA receptors on zebrafish M authner cells require CaMKII-alpha for normal development. Dev Neurobiol.

[65] Krapivinsky G, Krapivinsky L, Manasian Y, Ivanov A, Tyzio R, Pellegrino C (2003). The NMDA receptor is coupled to the ERK pathway by a direct interaction between NR2B and RasGRF1. Neuron.

[66] Sison M, Gerlai R (2011). Behavioral performance altering effects of MK-801 in zebrafish (*Danio rerio*). Behav Brain Res.

[67] Zimmermann FF, Gaspary KV, Siebel AM, Bonan CD (2016). Oxytocin reversed MK-801-induced social interaction and aggression deficits in zebrafish. Behav Brain Res.

[68] Myers SJ, Yuan H, Kang JQ, Tan FCK, Traynelis SF, Low CM (2019). Distinct roles of GRIN2A and GRIN2B variants in neurological conditions. F1000Res.

[69] XiangWei W, Jiang Y, Yuan H (2018). De novo mutations and rare variants occurring in NMDA receptors. Curr Opin Physiol.

[70] den Broeder MJ, van der Linde H, Brouwer JR, Oostra BA, Willemsen R, Ketting RF (2009). Generation and characterization of FMR1 knockout zebrafish. PLoS ONE.

[71] Marquez-Legorreta E, Constantin L, Piber M, Favre-Bulle IA, Taylor MA, Blevins AS (2022). Brain-wide visual habituation networks in wild type and fmr1 zebrafish. Nat Commun.

[72] Constantin L, Poulsen RE, Scholz LA, Favre-Bulle IA, Taylor MA, Sun B (2020). Altered brain-wide auditory networks in a zebrafish model of fragile X syndrome. BMC Biol.

[73] Wu YJ, Hsu MT, Ng MC, Amstislavskaya TG, Tikhonova MA, Yang YL (2017). Fragile X mental retardation-1 knockout zebrafish shows precocious development in social behavior. Zebrafish.

[74] Kim L, He L, Maaswinkel H, Zhu L, Sirotkin H, Weng W (2014). Anxiety, hyperactivity and stereotypy in a zebrafish model of fragile X syndrome and autism spectrum disorder. Prog Neuropsychopharmacol Biol Psychiatry.

[75] Cercato MC, Vazquez CA, Kornisiuk E, Aguirre AI, Colettis N, Snitcofsky M (2016). GluN1 and GluN2A NMDA receptor subunits increase in the hippocampus during memory consolidation in the rat. Front Behav Neurosci.

[76] Zimmermann FF, Gaspary KV, Leite CE, De Paula CG, Bonan CD (2015). Embryological exposure to valproic acid induces social interaction deficits in zebrafish (*Danio rerio*): a developmental behavior analysis. Neurotoxicol Teratol.

[77] Chen J, Lei L, Tian L, Hou F, Roper C, Ge X (2018). Developmental and behavioral alterations in zebrafish embryonically exposed to valproic acid (VPA): an aquatic model for autism. Neurotoxicol Teratol.

[78] Chomiak T, Turner N, Hu B (2013). What we have learned about autism spectrum disorder from valproic acid. Patholog Res Int.

[79] Christensen J, Gronborg TK, Sorensen MJ, Schendel D, Parner ET, Pedersen LH (2013). Prenatal valproate exposure and risk of autism spectrum disorders and childhood autism. JAMA.

[80] Ganz J, Kaslin J, Freudenreich D, Machate A, Geffarth M, Brand M (2012). Subdivisions of the adult zebrafish subpallium by molecular marker analysis. J Comp Neurol.

[81] Porter BA, Mueller T (2020). The zebrafish amygdaloid complex—functional ground plan, molecular delineation, and everted topology. Front Neurosci.

[82] Wullimann MF, Rink E (2002). The teleostean forebrain: a comparative and developmental view based on early proliferation, Pax6 activity and catecholaminergic organization. Brain Res Bull.

[83] Owens AP, Mathias CJ, Iodice V (2021). Autonomic dysfunction in autism spectrum disorder. Front Integr Neurosci.

[84] Levit-Binnun N, Davidovitch M, Golland Y (2013). Sensory and motor secondary symptoms as indicators of brain vulnerability. J Neurodev Disord.

[85] Suarez R, Gobius I, Richards LJ (2014). Evolution and development of interhemispheric connections in the vertebrate forebrain. Front Hum Neurosci.

[86] Petri Karl, Zhang Weiting, Ma Junyan, Schmidts Andrea, Lee Hyunho, Horng Joy E., Kim Daniel Y., Kurt Ibrahim C., Clement Kendell, Hsu Jonathan Y., Pinello Luca, Maus Marcela V., Joung J. Keith, Yeh Jing-Ruey Joanna (2022). CRISPR prime editing with ribonucleoprotein complexes in zebrafish and primary human cells. Nature Biotechnology.

