# Supplementary material for: Disruption of grin2B, an ASD-associated gene, produces social deficits in zebrafish
Source: Mol Autism. 2022 Sep 22;13:38. doi: 10.1186/s13229-022-00516-3 (PMC9502958; doi:10.1186/s13229-022-00516-3)
Supplement: Supplementary file 1 — Additional file 1. Supplementary Figures 1–8 and Supplementary Tables 1–5: Figure S1. Alignments of hGluN2B, zGluN2Ba and zGluN2Bb. Table S1. Social behavior for grin2B-/- fish. Figure S2. grin2B single mutants do not show a juvenile social deficit. Table S2. Social behavior for grin2B single mutant fish. Figure S3. Wild-type fish show no preference for social interactions with wild-type when compared to grin2B-/-. Table S3. Social behavior with grin2B-/- as conspecific. Table S4. Social behavior for an ASD-associated gene and other NMDAR subunits. Figure S4. Generation of loss-of-function lesions in grin2A and grin2D subunit paralogues. Figure S5. grin2B-/- fish show wild-type spontaneous and photic evoked responses throughout early larval stages. Figure S6. grin2B-/- have normal swim behavior at 3 or 4wpf. Figure S7. grin2B single mutants do not show a larval feeding deficit. Figure S8. Zebrafish subpallium schematic. Table S5. Proposed mammalian homologies for zebrafish subpallial nuclei. [file 13229_2022_516_MOESM1_ESM.docx]

ADDITIONAL FILE

**Disruption of *grin2B*, an ASD-associated gene, produces social deficits in zebrafish.**

**Zoodsma et al.**

Additional file 1: Fig. S1. Alignments of hGluN2B, zGluN2Ba and zGluN2Bb (Relates to Figure 1) 2

Additional file 1: Table S1. Social behavior for *grin2B^-/-^* fish (Relates to Figure 3) 3

Additional file 1: Fig. S2. *grin2B* single mutants do not show a juvenile social deficit (Relates to Figure 3) 3

Additional file 1: Table S2. Social behavior for *grin2B* single mutant fish (Relates to Supplemental Figure 2) 4

Additional file 1: Fig. S3. Wild-type fish show no preference for social interactions with wild-type when compared to *grin2B^-/-^* (Relates to Figure 3) 5

Additional file 1: Table S3. Social behavior with *grin2B^-/-^* as conspecific (Relates to Supplemental Figure 3) 6

Additional file 1: Table S4. Social behavior for an ASD-associated gene and other NMDAR subunits (Relates to Figure 4) 7

Additional file 1: Fig. S4. Generation of loss-of-function lesions in *grin2A* and *grin2D* subunit paralogues (relates to Figure 4). 8

Additional file 1: Fig. S5. *grin2B*^-/-^ fish show wild-type spontaneous and photic evoked responses throughout early larval stages (relates to Figure 5). 10

Additional file 1: Fig. S6. *grin2B^-/-^* have normal swim behavior at 3 or 4wpf (relates to Figure 3 and 5). 11

Additional file 1: Fig. S7. *grin2B* single mutants do not show a larval feeding deficit (Relates to Figure 5) 11

Additional file 1: Fig. S8. Zebrafish subpallium schematic (Relates to Figure 7 & Supplementary Table 5) 12

Additional file 1: Table S5. Proposed mammalian homologies for zebrafish subpallial nuclei (Relates to Figure 7) 13


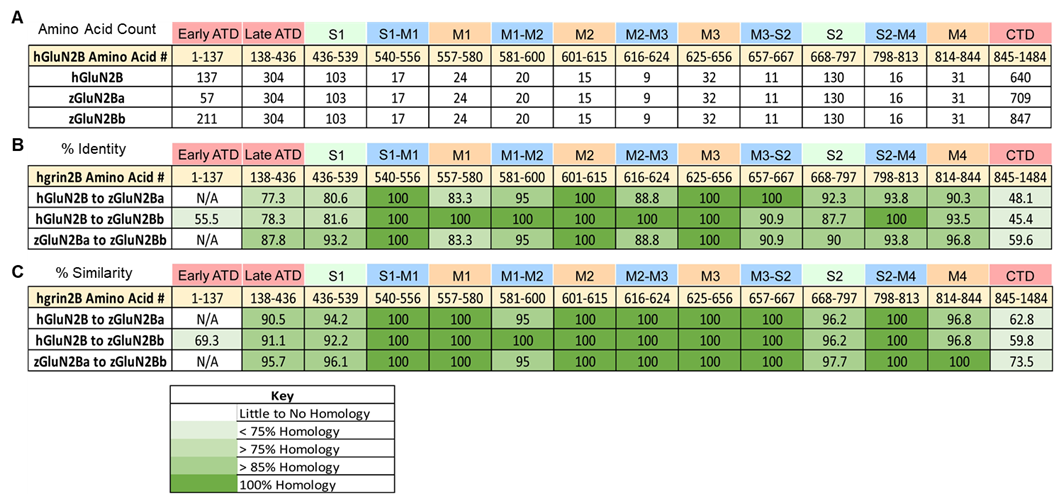


# Additional file 1: Fig. S1. Alignments of hGluN2B, zGluN2Ba and zGluN2Bb (Relates to Figure 1)

**(A)** hGluN2B divided into 14 distinct sequential structural regions: Early and late ATD (Amino terminal domain), 2 domains that make up the ligand binding domain (S1 and S2), 3 transmembrane segments (M1, M3 and M4) and an M2 pore loop, 5 TMD-associated linkers (S1-M1, M1-M2, M2-M3, M3-S2, and S2-M4), and the CTD (Carboxyl terminal domain). Table shows number of amino acids in each domain for hGluN2B, and both zebrafish proteins (zGluN2Ba and zGluN2Bb). Note identical amino acid numbers for each region except the ATD and CTD for all proteins.

**(B and C)** Amino acid composition homology between hGluN2B and both zebrafish proteins (zGluN2Ba and zGluN2Bb). **(B)** indicates percentage identity while **(C)** indicates the percentage similarity of the amino acid sequences. Both zebrafish paralogues show high similarity to each other and to hGluN2B.

Key denoting color for percent homology of each domain.

# Additional file 1: Table S1. Social behavior for *grin2B^-/-^* fish (Relates to Figure 3)

| **Age of Zebrafish** | **Genotype** | **SPI** | **n** | **p-value** |
| --- | --- | --- | --- | --- |
| 1 wpf | Wild-type *grin2B^-/-^* | 0.15 ± 0.07 0.02 ± 0.07 | 29 23 | 0.66 |
| 2 wpf | Wild-type *grin2B^-/-^* | 0.15 ± 0.04 0.10 ± 0.02 | 29 26 | 0.99 |
| 3 wpf | Wild-type *grin2B^-/-^* | 0.41 ± 0.05 0.15 ± 0.02 | 28 24 | 0.02* |
| 4 wpf | Wild-type *grin2B^-/-^* | 0.58 ± 0.08 0.24 ± 0.04 | 24 23 | 9.2e-4*** |

Values shown are mean ± SEM. *grin2B^-/-^* fish were generated from homozygous mutant intercrosses. All p-values are from a 2-factor ANOVA and post-hoc Tukey test with age of fish and genotype as the two factors.

*(*p < 0.05, **p < 0.01, or ***p < 0.001*)


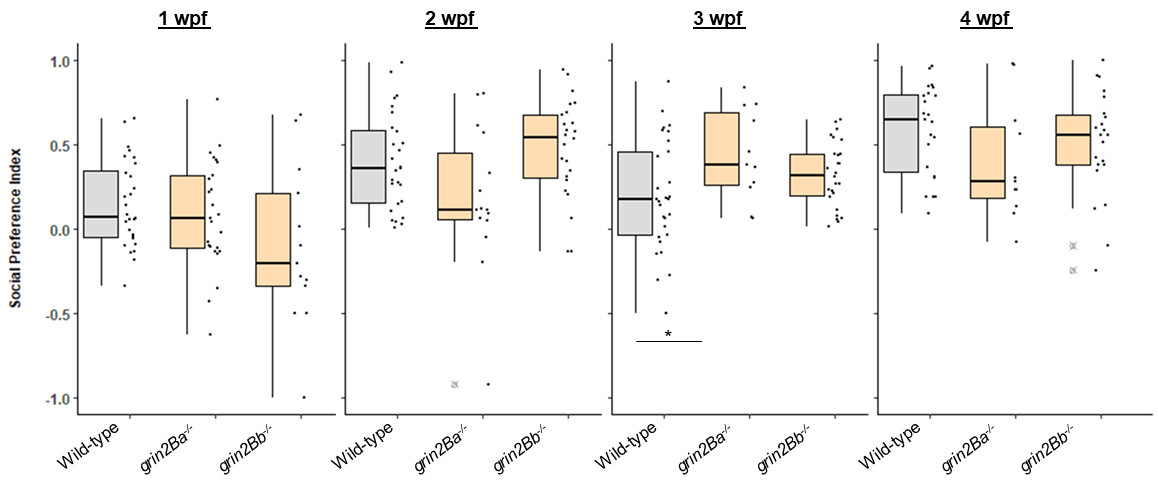


# Additional file 1: Fig. S2. *grin2B* single mutants do not show a juvenile social deficit (Relates to Figure 3)

Social Preference Index (SPI) for *grin2B* single mutants assayed weekly from 1 to 4 wpf. Experimental details in Supplementary Table 2

# Additional file 1: Table S2. Social behavior for *grin2B* single mutant fish (Relates to Supplemental Figure 2)

| **Age of Zebrafish** | **Genotype** | **SPI** | **n** | **p-value (*Test*)** |
| --- | --- | --- | --- | --- |
| 1 wpf | Wild-type *grin2Ba^-/-^ grin2Bb^-/-^* | 0.14 ± 0.05 0.08 ± 0.06 -0.10 ± 0.13 | 28 25 13 | 0.09 (*1-Factor ANOVA*) |
| 2 wpf | Wild-type *grin2Ba^-/-^ grin2Bb^-/-^* | 0.40 ± 0.06 0.26 ± 0.08 0.48 ± 0.06 | 27 14 24 | 0.10  (*1-Factor ANOVA*) |
| 3 wpf | Wild-type *grin2Ba^-/-^ grin2Bb^-/-^* | 0.19 ± 0.06 0.44 ± 0.08 0.32 ± 0.04 | 29 11 26 | 0.034 * (*1-Factor ANOVA*) |
| 4 wpf | Wild-type *grin2Ba^-/-^ grin2Bb^-/-^* | 0.58 ± 0.06 0.39 ± 0.10 0.57 ± 0.05 | 22 10 19 | 0.28 (*1-Factor ANOVA*) |

Values shown are mean ± SEM. All fish were generated from homozygous intercrosses.
A Tukey post-Hoc test was run on all groups with a p-value < 0.05. 3 wpf comparison Post-Hoc Tukey test :
Wild-type to *grin2Ba^-/-^*: p = 0.037*
Wild-type to *grin2Bb^-/-^*: p = 0.20
*grin2Ba^-/-^* to *grin2Bb^-/-^*: p = 0.47
*(*p < 0.05, **p < 0.01, or ***p < 0.001*)


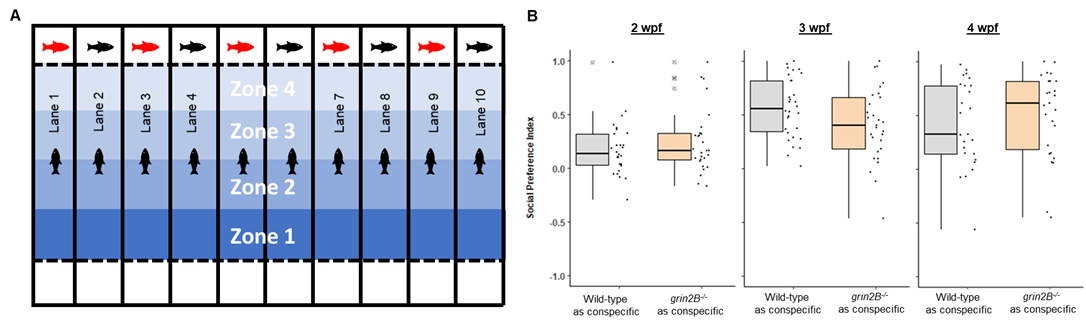


# Additional file 1: Fig. S3. Wild-type fish show no preference for social interactions with wild-type when compared to *grin2B^-/-^* (Relates to Figure 3)

**(A)** Schematic depiction of social behavior chamber. Each lane contains a wild-type fish (depicted as a black fish). At the end of each chamber is a clear glass window that is empty on one side and contains either a wild-type age-matched conspecific or a *grin2B^-/-^* (here indicated by a red fish) behind it. Each chamber is sub-divided into four arbitrary zones, with Zone 4 designated the zone closest to the conspecific.

**(B)** Wild-type zebrafish show no decreased association with *grin2B*^-/-^ as compared to wild-type conspecifics. SPI was calculated at successive weeks for the experiment outlined in (**A**) Fish were assayed weekly from 2 to 4 wpf with experimental details in **Table 3**.

# Additional file 1: Table S3. Social behavior with *grin2B^-/-^* as conspecific (Relates to Supplemental Figure 3)

Values shown are mean ± SEM. All test fish (freely swimming the length of the lane) in this assay are wild type. Conspecific genotype refers to the genotype of the fish in the small chamber directly above Zone 4. *grin2B^-/-^* were generated from double homozygous mutant intercrosses.

*(*p < 0.05, **p < 0.01, or ***p < 0.001*)

# Additional file 1: Table S4. Social behavior for an ASD-associated gene and other NMDAR subunits (Relates to Figure 4)

| **Gene** | **Genotype** | **Cross to Generate** | **SPI** | **n** | **p-value (Test)** |
| --- | --- | --- | --- | --- | --- |
| *fmr1* | *fmr1^+/+^* *fmr1^+/-^ fmr1^-/-^* | Heterozygous intercross | 0.46 ± 0.08 0.15 ± 0.03 0.25 ± 0.05 | 10 33 15 | 4.1e-4***  (*1-Factor ANOVA*) |
| *grin1a* | *grin1a^+/+^* *grin1a^+/-^* *grin1a^-/-^* | Heterozygous intercross | 0.33 ± 0.10 0.36 ± 0.05 0.42 ± 0.07 | 10 38 16 | 0.74  (*1-Factor ANOVA*) |
| *grin1b* | *grin1b^+/+^* *grin1b^-/-^* | Homozygote intercross Homozygote intercross | 0.58 ± 0.06 0.50 ± 0.04 | 29 28 | 0.31 (*t-test*) |
| *grin2A* | *grin2Aa^+/+^ grin2Aa^+/-^; grin2Ab^-/-^*  *grin2Aa^-/-^; grin2Ab^-/-^* | Homozygous intercross *(grin2Aa^+/-^; grin2Ab^-/-^)* crossed to *(grin2Aa^-/-^; grin2Ab^-/-^)* | 0.37 ± 0.08 0.42 ± 0.06  0.61 ± 0.06 | 25 20  15 | 0.09 (*1-Factor ANOVA*) |
| *grin2Da* | *grin2Da^+/+^* *grin2Da^+/-^* *grin2Da^-/-^* | Heterozygous intercross | 0.39 ± 0.12 0.37 ± 0.06 0.32 ± 0.08 | 1236 16 | 0.43  (*1-Factor ANOVA*) |
| *grin2Db* | *grin2Db^+/+^* *grin2Db^+/-^* *grin2Db^-/-^* | Heterozygous intercross | 0.43 ± 0.11 0.41 ± 0.07 0.55 ± 0.06 | 16 25 21 | 0.43  (*1-Factor ANOVA*) |

Values shown are mean ± SEM. All assays were run at 3 wpf. *( ***p < 0.001*)
A Tukey post-Hoc test was run on all groups with a p-value < 0.05. 3 wpf comparison Post-Hoc Tukey test :
*fmr1^+/+^* to *fmr1^+/-^*: p = 2.6e-4***
*fmr1^+/+^* to *fmr1^-/-^*: p = 0.037*
*fmr1^+/-^* to *fmr1^-/-^*: p = 0.27


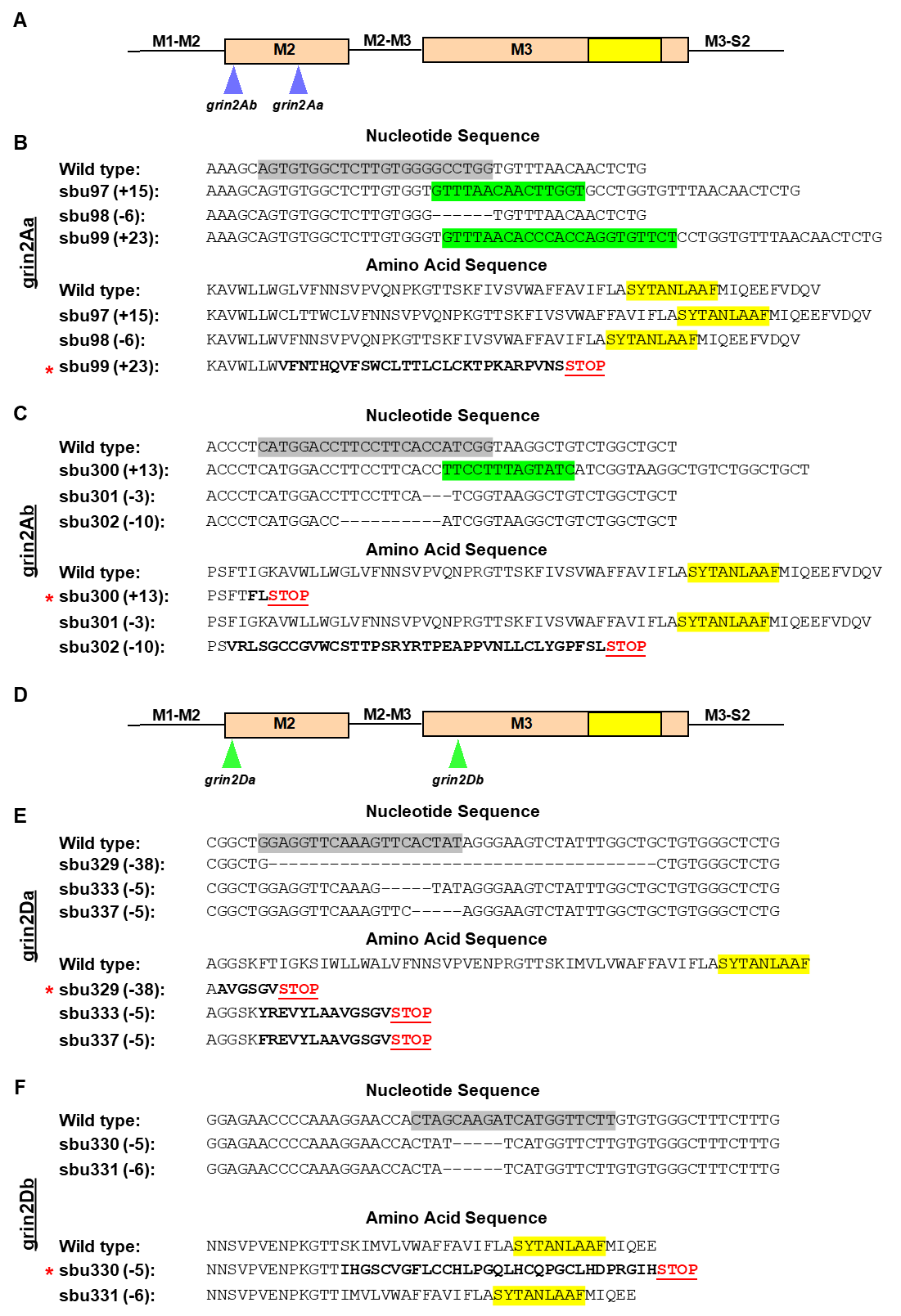


# Additional file 1: Fig. S4. Generation of loss-of-function lesions in *grin2A* and *grin2D* subunit paralogues (relates to Figure 4).

**(A & D)** Linear representation of the M2 and M3 segments indicating gRNA target site for **(A)** *grin2Aa* and *grin2Ab* (blue arrows) or the **(D)** *grin2Da* and *grin2Db* (green arrows). The SYTANLAAF motif (indicated in yellow) is the most highly conserved motif in iGluRs and is fundamental to their function.

**(B-C & E-F)** Alignments of nucleotide and amino acid sequences for **(B)** *grin2Aa,* **(C)** *grin2Ab,* **(E)** *grin2Da* and **(F)** *grin2Db*. Induced mutations within the nucleotide sequences are denoted as either dashes (deletions) or highlighted green (insertions). gRNA target site on the nucleotide sequence (gray highlight) are adjacent to generated mutations. Altered amino acid sequence (bolded) and early stop codons (Red STOP) are generated in sbu99 for *grin2Aa*; sbu300 for *grin2Ab;* sbu329, sbu333 and sbu337 for *grin2Da;* and sbu330 for *grin2Db*. Such early translation termination events would prevent encoding the SYTANLAAF motif (yellow highlight) as well as the D2 lobe of the LBD, which would make the receptor non-functional. Red asterisk indicate the preferred allele for each gene (utilized in **Figure 4**), which has the earliest induced stop codon.


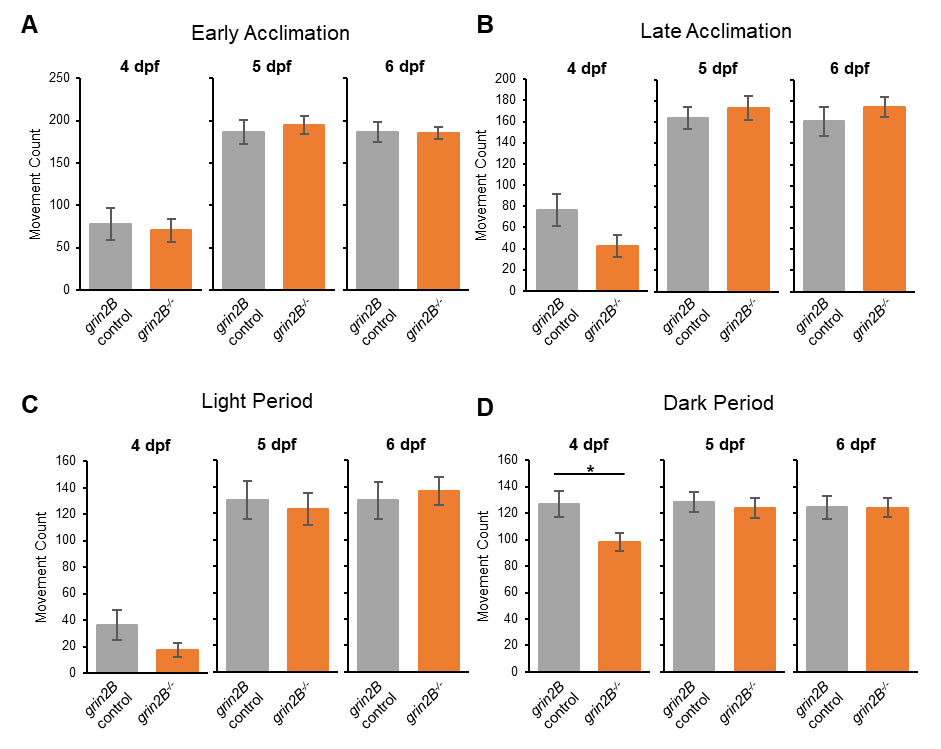


# Additional file 1: Fig. S5. *grin2B*^-/-^ fish show wild-type spontaneous and photic evoked responses throughout early larval stages (relates to Figure 5).

**(A-D)** Spontaneous and photic evoked larval swim behavior assay. Bar graphs of average movement count in **(A)** early acclimation, **(B)** late acclimation, **(C)** spontaneous movement in the light and **(D)** movement after the removal of illumination as outlined in the social behavior paradigm in **(Fig. 5)**. Zebrafish larvae at 4, 5 and 6 dpf were tested in 24-well dishes; *grin2B* control (n = 19, 22, 22) and *grin2B*^-/-^ fish (n = 27, 31, 31) for 4, 5, and 6 dpf respectively. (*p = 0.024*, t-test)*


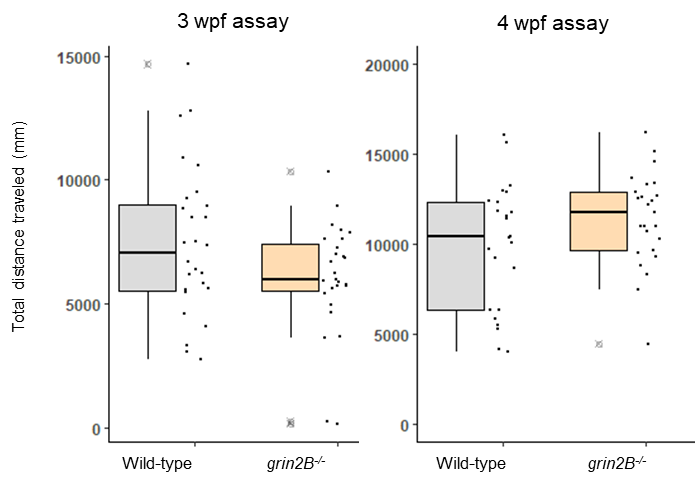


# Additional file 1: Fig. S6. *grin2B^-/-^* have normal swim behavior at 3 or 4wpf (relates to Figure 3 and 5).

*grin2B^-/-^* fish show similar swim activity (measured in distance traveled) over the course of the social behavior assay (20 minutes of recorded movement) at both 3 (n = 28, 24) and 4 (n = 24, 23) wpf for wild-type and *grin2B^-/-^* fish respectively. Data points here are the mean travel distance over the entire 20 minutes of recording for individual fish in both genotype


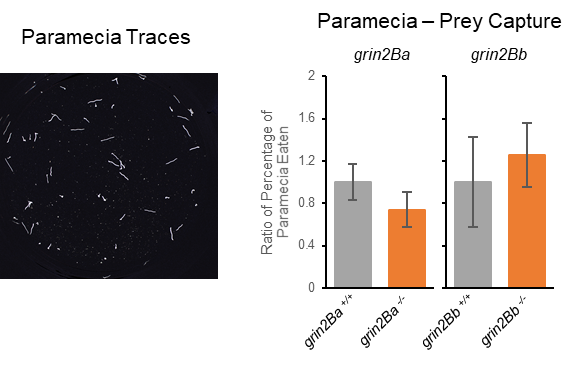


# Additional file 1: Fig. S7. *grin2B* single mutants do not show a larval feeding deficit (Relates to Figure 5)

Proportion of paramecia eaten over the trial period (mean ± SEM) normalized to control for *grin2B* single mutants ((n = 12, 16) for *grin2Ba^+/+^* and *grin2Ba^-/-^* respectively and (n = 26, 12) for *grin2Bb^+/+^* and *grin2Bb^-/-^* respectively)


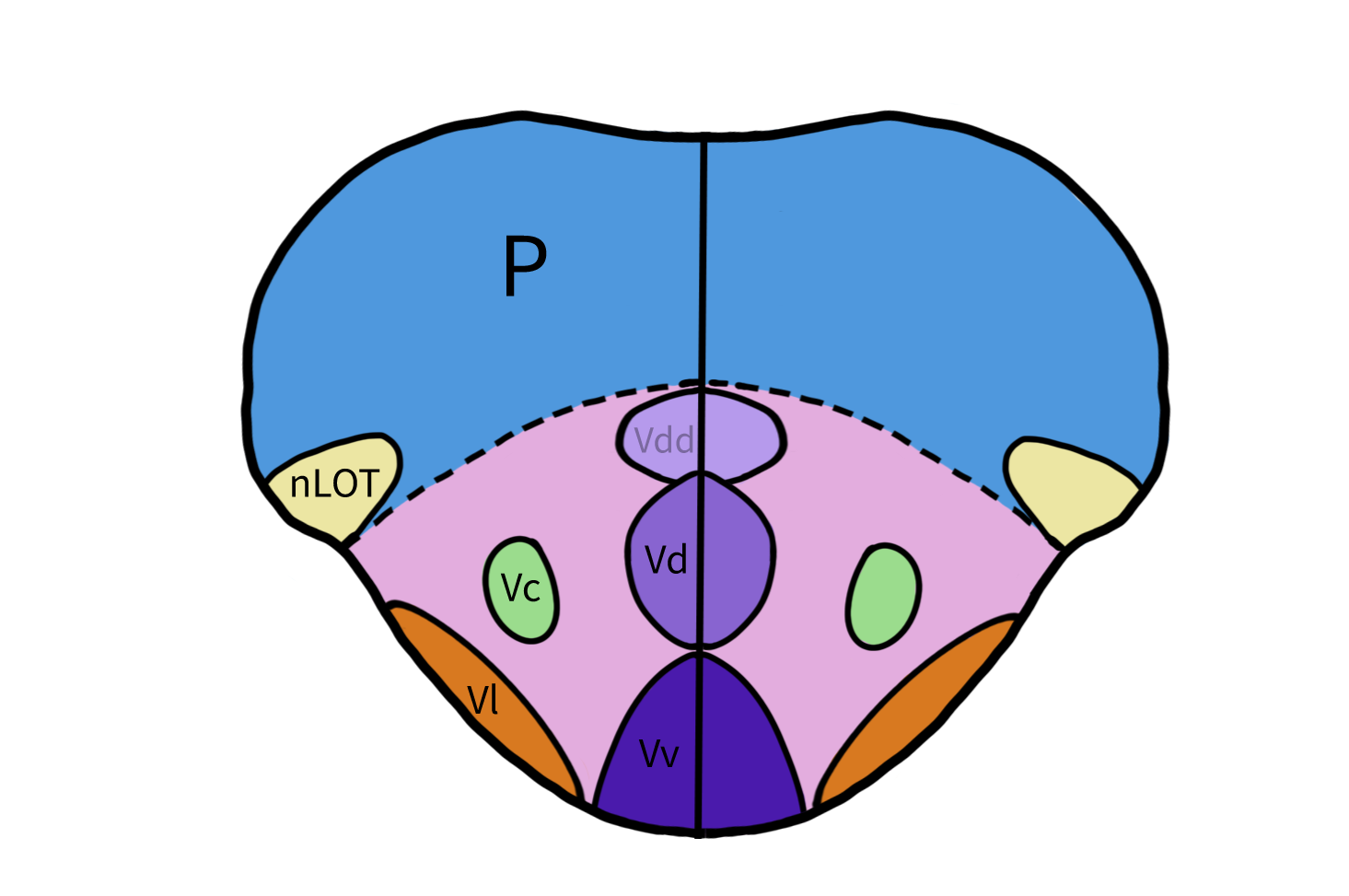


# Additional file 1: Fig. S8. Zebrafish subpallium schematic (Relates to Figure 7 & Supplementary Table 5)

Schematic outlining distinct subpallial regions in the zebrafish. P (pallium), nLOT (Nucleus of the Lateral Olfactory Tract), Vl (Lateral Subpallium), Vv (Ventromedial Subpallium), Vc (Ventrolateral Subpallium), Vd (Dorsomedial Subpallium) and Vdd (Proposed dorsal-most Dorsomedial Subpallium)

# Additional file 1: Table S5. Proposed mammalian homologies for zebrafish subpallial nuclei (Relates to Figure 7)

| **Anatomical Location** | **Zebrafish Nuclei** | **Homologous Mammalian Structure** | **Functional System** |
| --- | --- | --- | --- |
| Dorsomedial Subpallium | Vd ^1,4^ | Striatum | Basal Ganglia |
| Ventromedial Subpallium (Dorsal aspect) | Vv ^1^ | Pallidum | Basal Ganglia |
| Ventromedial Subpallium (Ventral aspect) | Vv^3^ | Septum | Limbic System |
| Lateral Subpallium | Vl ^4^ | Septal Nuclei and  Ventral Striatum | Limbic System and Basal Ganglia |
| Dorsomedial subpallium | Vdd ^2^ | Central Amygdala  anterior division (CeAa) | Amygdala |
| Ventrolateral subpallium | Vc ^2^ | Central Amygdala  lateral division (CeAl) | Amygdala |
| Pallial, subpallial, and thalamic derivatives | nLOT ^2^ | Nucleus of the  Lateral Olfactory Tract | Lateral Olfactory Tract (Amygdala input tract) |

^1^ ([Ganz et al., 2012](#_ENREF_1))
^2^ ([Porter & Mueller, 2020](#_ENREF_3))
^3^ ([Wullimann & Rink, 2002](#_ENREF_4))
^4^ ([Mueller, Vernier, & Wullimann, 2004](#_ENREF_2))

Subpallial zebrafish nuclei subdivisions and homologies are not fully resolved. This table outlines the generally agreed upon homologies based on the indicated sources.

**References for Additional File**

Ganz, J., Kaslin, J., Freudenreich, D., Machate, A., Geffarth, M., & Brand, M. (2012). Subdivisions of the adult zebrafish subpallium by molecular marker analysis. *J Comp Neurol, 520*(3), 633-655. doi:10.1002/cne.22757

Mueller, T., Vernier, P., & Wullimann, M. F. (2004). The adult central nervous cholinergic system of a neurogenetic model animal, the zebrafish Danio rerio. *Brain Res, 1011*(2), 156-169. doi:10.1016/j.brainres.2004.02.073

Porter, B. A., & Mueller, T. (2020). The Zebrafish Amygdaloid Complex - Functional Ground Plan, Molecular Delineation, and Everted Topology. *Front Neurosci, 14*, 608. doi:10.3389/fnins.2020.00608

Wullimann, M. F., & Rink, E. (2002). The teleostean forebrain: a comparative and developmental view based on early proliferation, Pax6 activity and catecholaminergic organization. *Brain Res Bull, 57*(3-4), 363-370. doi:10.1016/s0361-9230(01)00666-9
